# Changes in Ploidy Drive Reproduction Transition and Genomic Diversity in a Polyploid Fish Complex

**DOI:** 10.1093/molbev/msac188

**Published:** 2022-09-03

**Authors:** Meng Lu, Zhi Li, Zi-Yu Zhu, Fang Peng, Yang Wang, Xi-Yin Li, Zhong-Wei Wang, Xiao-Juan Zhang, Li Zhou, Jian-Fang Gui

**Affiliations:** State Key Laboratory of Freshwater Ecology and Biotechnology, Hubei Hongshan Laboratory, the Innovation Academy of Seed Design, Institute of Hydrobiology, Chinese Academy of Sciences, Wuhan 430072, China; University of Chinese Academy of Sciences, Beijing 100049, China; State Key Laboratory of Freshwater Ecology and Biotechnology, Hubei Hongshan Laboratory, the Innovation Academy of Seed Design, Institute of Hydrobiology, Chinese Academy of Sciences, Wuhan 430072, China; University of Chinese Academy of Sciences, Beijing 100049, China; State Key Laboratory of Freshwater Ecology and Biotechnology, Hubei Hongshan Laboratory, the Innovation Academy of Seed Design, Institute of Hydrobiology, Chinese Academy of Sciences, Wuhan 430072, China; University of Chinese Academy of Sciences, Beijing 100049, China; State Key Laboratory of Freshwater Ecology and Biotechnology, Hubei Hongshan Laboratory, the Innovation Academy of Seed Design, Institute of Hydrobiology, Chinese Academy of Sciences, Wuhan 430072, China; University of Chinese Academy of Sciences, Beijing 100049, China; State Key Laboratory of Freshwater Ecology and Biotechnology, Hubei Hongshan Laboratory, the Innovation Academy of Seed Design, Institute of Hydrobiology, Chinese Academy of Sciences, Wuhan 430072, China; University of Chinese Academy of Sciences, Beijing 100049, China; State Key Laboratory of Freshwater Ecology and Biotechnology, Hubei Hongshan Laboratory, the Innovation Academy of Seed Design, Institute of Hydrobiology, Chinese Academy of Sciences, Wuhan 430072, China; University of Chinese Academy of Sciences, Beijing 100049, China; State Key Laboratory of Freshwater Ecology and Biotechnology, Hubei Hongshan Laboratory, the Innovation Academy of Seed Design, Institute of Hydrobiology, Chinese Academy of Sciences, Wuhan 430072, China; University of Chinese Academy of Sciences, Beijing 100049, China; State Key Laboratory of Freshwater Ecology and Biotechnology, Hubei Hongshan Laboratory, the Innovation Academy of Seed Design, Institute of Hydrobiology, Chinese Academy of Sciences, Wuhan 430072, China; University of Chinese Academy of Sciences, Beijing 100049, China; State Key Laboratory of Freshwater Ecology and Biotechnology, Hubei Hongshan Laboratory, the Innovation Academy of Seed Design, Institute of Hydrobiology, Chinese Academy of Sciences, Wuhan 430072, China; University of Chinese Academy of Sciences, Beijing 100049, China; State Key Laboratory of Freshwater Ecology and Biotechnology, Hubei Hongshan Laboratory, the Innovation Academy of Seed Design, Institute of Hydrobiology, Chinese Academy of Sciences, Wuhan 430072, China; University of Chinese Academy of Sciences, Beijing 100049, China

**Keywords:** polyploid *Carassius* complex, unisexual reproduction, genomic decay, reproduction transition, meiosis, clonal diversity

## Abstract

Unisexual animals are commonly found in some polyploid species complexes, and most of these species have had a long evolutionary history. However, their method for avoiding genomic decay remains unclear. The polyploid *Carassius* complex naturally comprises the sexual amphidiploid *C. auratus* (crucian carp or goldfish) (AABB) and the gynogenetic amphitriploid *C. gibelio* (gibel carp) (AAABBB). Recently, we developed a fertile synthetic amphitetraploid (AAAABBBB) male from *C. gibelio* by incorporating a *C. auratus* genome. In this study, we generated novel amphitriploids (AAABBB) by backcrossing the amphitetraploid male with the amphidiploid *C. auratus*. Whole-genome resequencing revealed the genomic changes, including recombination and independent assortment between homologs of *C. gibelio* and *C. auratus*. The fertility, sex determination system, oocyte development, and fertilization behaviors of the novel amphitriploids were investigated. Approximately 80% of the novel amphitriploid females recovered the unisexual gynogenesis ability. Intriguingly, two types of primary oocyte (with and without homolog synapsis) were discovered, and their distinct development fates were observed. Type I oocytes entered apoptosis due to improper synaptonemal complex assembly and incomplete double-strand break repair, whereas subsequent type II oocytes bypassed meiosis through an alternative ameiotic pathway to develop into mature eggs. Moreover, gynogenesis was stabilized in their offspring, and a new array of diverse gynogenetic amphitriploid clones was produced. These revealed genomic changes and detailed cytological data provide comprehensive evidence that changes in ploidy drive unisexual and sexual reproduction transition, thereby resulting in genomic diversity and allowing *C. gibelio* avoid genomic decay.

## Introduction

The origin and maintenance of unisexual vertebrates have long fascinated evolutionary biologists, and unisexual animals are considered ideal systems for addressing some long-standing and fundamental questions in ecology and evolution (Gui and Zhou 2010; Van de Peer et al. 2017; Brunes et al. 2019; Laskowski et al. 2019; Gui et al. 2022; Li et al. 2022). Over 100 species or biotypes across around 22 genera of fish, amphibians, and reptiles are able to reproduce via parthenogenesis, gynogenesis, or hybridogenesis (Gui and Zhou 2010; Avise 2015; Zhou and Gui 2017). However, as an evolutionary genetic paradox, the unusual evolutionary persistence, clonal diversity, and reproductive strategy of unisexual vertebrates remain a puzzle (Hojsgaard and Schartl 2021).

Most unisexual vertebrate species or biotypes arose via hybridization or polyploidization (Avise 2015), and the majority (>60%) are polyploids (Vrijenhoek 1989; Mateos and Vrijenhoek 2005). These species usually live in sympatric association with their bisexual diploid relatives and form sexual diploid–unisexual polyploid complexes, such as the gynogenetic *Poeciliopsis monacha*-*lucida* complex (Mateos and Vrijenhoek 2005), the parthenogenetic *Cobitis elongatoides*-*taenia* complex (Janko et al. 2003), the parthenogenetic *Loxopholis percarinatum* complex (Brunes et al. 2019), the gynogenetic *Ambystoma laterale*-*jeffersonianum* complex (Bogart 2019), and the gynogenetic *Carassius* complex (Gui and Zhou 2010). Traditionally, unisexual lineages have been predicted to be evolutionarily short-lived by classical theories, such as the Red Queen hypothesis (Bell 1982) and Muller’s ratchet model (Muller 1964). Due to the absence of normal meiosis, clonal lineages lack recombinational genetic variation and accumulate deleterious mutations, which inevitably lead to genomic decay (Gui and Zhou 2010; Avise 2015; Hojsgaard and Schartl 2021). However, some well-known unisexual vertebrates have evolved for more than several hundred thousand years and have successfully colonized wider and harsher habitats than their sexual relatives (Spolsky et al. 1992; Lampert and Schartl 2008; Loewe and Lamatsch 2008; Pellino et al. 2013; Brunes et al. 2019). For example, molecular dating supports an ancient origin of unisexual *Ambystoma* at the early Pliocene (∼5 My), a longer evolutionary history than expected (Bi and Bogart 2010). The Amazon molly (*Poecilia formosa*), the first described unisexual vertebrate (Hubbs and Hubbs 1932), may have existed for around 100,000 years (Warren et al. 2018), with ∼500,000 generations, which is beyond the theoretical extinction time (104–105 generations) of a strictly clonal vertebrate population (Gabriel 1990). Surprisingly, but significantly, the genome analysis indicates that the Amazon molly is in remarkably good “genomic health” (Samani and Reuter 2018), showing little genetic decay, as well as high diversity and heterozygosity (Warren et al. 2018). Therefore, these ancient unisexual vertebrates have intrigued evolutionary biologists and inspired investigations into how deleterious mutations can be purged and how sufficient genetic diversity can be generated in unisexual organisms (Gui and Zhou 2010; Avise 2015).

Unisexual organisms have developed various strategies to conquer the negative consequences of meiotic absence, such as intergenomic recombination (Bi and Bogart 2006; Barley et al. 2022), genome replacement (Bogart 2019), and sporadic recombination or gene conversion (Warren et al. 2018; Wang, Li, Xu, et al. 2022). Moreover, accumulating evidence shows that the borderline between sexuality and unisexuality is blurred and these factors seem to exist on a continuum, ranging from meiotic sex with recombination, to more or less distorted meiotic divisions, to completely ameiotic development (Stenberg and Saura 2013; Janko et al. 2021). The reproduction transition between sexuality and unisexuality has been described in several species complexes, such as the dandelion *Taraxacum* complex (Van Dijk et al. 2003, 2016; Verduijn et al. 2004), the *Cobitis hankugensis-longicorpus* complex (Saitoh et al. 2004; Kwan et al. 2019), and the Iberian cyprinid *Squalius alburnoides* complex (Alves et al. 2001; Crespo-López et al. 2006). The reproduction transition switch has been suggested to lead to genetic exchange between sexual and unisexual populations, but the genomic changes and cytological mechanisms underlying the reproduction transition remain unclear.

The polyploid *Carassius* species complex naturally comprises the sexual *C. auratus*, which has 100 chromosomes, and the gynogenetic *C. gibelio* and *C. langsdorfi*, which each have about 150 chromosomes (Cherfas 1981; Jiang et al. 1983; Takai and Ojima 1983). Previous phylogenetic studies have revealed that gynogenetic *C. gibelio* and *C. langsdorfi* may have undergone multiple independent autotriploidy events from sympatric *C. auratus* (Takada et al. 2010; Gao et al. 2012; Luo et al. 2014; Liu, Li, et al. 2017; Liu, Jiang, et al. 2017). *Carassius gibelio* was demonstrated to be able to reproduce by gynogenesis when its eggs were fertilized by sperm from sexual relative species (Cherfas 1981; Jiang et al. 1983), and many diverse gynogenetic clones and a minor proportion (1.2–26.5%) of males have been found in natural populations of *C. gibelio* (Jiang et al. 2013; Liu, Li, et al. 2017; Li et al. 2018). In our early study, several DNA fragments inherited from paternal clone D were identified in the offspring of a *C. gibelio* clone F (♀) × clone D (♂) by rapid amplified polymorphic DNA analysis, implying that *C. gibelio* may reproduce bisexually (Zhou et al. 2000). Recently, we discovered that *C. gibelio* has both a genotypic sex determination (GSD) system and a temperature-dependent sex determination system (Li et al. 2016a, 2018). GSD is closely associated with male-specific supernumerary microchromosomes (Li et al. 2016a; Ding et al. 2021). Moreover, we found that a variant mode of gynogenesis, not sexual reproduction, is initiated when *C. gibelio* females are mated with their genotypic males (GSD males). This variant of gynogenesis leads to the occurrence of GSD males and an increase in genetic diversity (Zhao et al. 2021), which may be associated with the inheritance of the paternal DNA fragments from *C. gibelio* males observed in our previous study (Zhou et al. 2000; Zhao et al. 2021). Significantly, kleptogenesis or allogynogenesis, referring to the introgression of paternal genetic material from sexual relative species into gynogenetic offspring, has been suggested to contribute to the genetic diversity of some unisexual species, such as Amazon molly, unisexual Ambystoma, and *C. gibelio* (Jiang et al. 1983; Schartl et al. 1995; Bogart et al. 2007; Warren et al. 2018; Bogart 2019; Chen et al. 2020). Occasionally, a few individuals with higher ploidy, arising from accidental incorporation of a sperm nucleus from a sexual donor into a gynogenetic egg, were discovered in natural or reared populations of the *Carassius* complex (Takai and Ojima 1983; Gui et al. 1993; Li et al. 2016b; Lu et al. 2018), and some of these males were found to produce diploid sperm (Dong et al. 2013; Lu et al. 2021; Yamaguchi et al. 2021). Although it has been assumed that these males contribute to the diversification of gynogenetic *Carassius* (Lu et al. 2021; Mishina et al. 2021), the detailed biological process and the underlying mechanisms remain unknown.

Recent genome studies have suggested that *C. auratus* likely originated from an allotetraploidy event (Chen et al. 2019; Kon et al. 2020; Luo et al. 2020). To study the evolutionary mechanisms, we have comparatively sequenced the genomes of *C. auratus* and *C. gibelio*, and assembled their haplotypes to chromosome level, indicating that they have identical A and B subgenomes. Relative to the AABB amphidiploid (Lawrence and Pikaard 2003) characteristics of *C. auratus*, *C. gibelio* has been revealed to be amphitriploid (AAABBB), with two triploid sets of chromosomes (Wang, Li, Xu, et al. 2022). Intriguingly, we further identified a fertile male from a group of synthetic amphitetraploids (∼200 chromosomes, SA4n) that incorporated one amphidiploid *C. auratus* genome (50 chromosomes) into amphitriploid *C. gibelio* (∼150 chromosomes) (Lu et al. 2021). In addition, the known X/Y sex determination system (Yamamoto and Kajishima 1968; Wen et al. 2020) was transferred from sexual *C. auratus* to the amphitetraploid, and some amphitetraploid males regained sexual reproduction ability (Lu et al. 2021), providing a rare opportunity to explore the effects of ploidy change and reproduction transition on genetic and clonal diversity in the polyploid *Carassius* species complex.

In the current study, we first generated a group of novel amphitriploids by backcrossing the fertile synthetic amphitetraploid male with *C. auratus* females, and demonstrated their divergent amphitriploid characteristics with high genetic diversity resulting from sexual reproduction. Subsequently, we applied whole-genome resequencing and chromosomal genotyping to analyze their diverse chromosomal compositions and significant genomic changes. Then, we utilized coimmunostaining on oocyte microspreads to show the oocyte development and fate. Finally, diverse gynogenetic amphitriploid clones with abundant genetic diversity were reproduced by the unisexual–sexual–unisexual reproduction transition. The current study uncovers the genomic changes and cytological mechanisms underlying ploidy change and reproduction transition, and provides novel insights into the reproduction strategy for unisexual animals to avoid genomic decay.

## Results

### High Genetic Diversity in Novel Amphitriploids

**Fig. 1.**
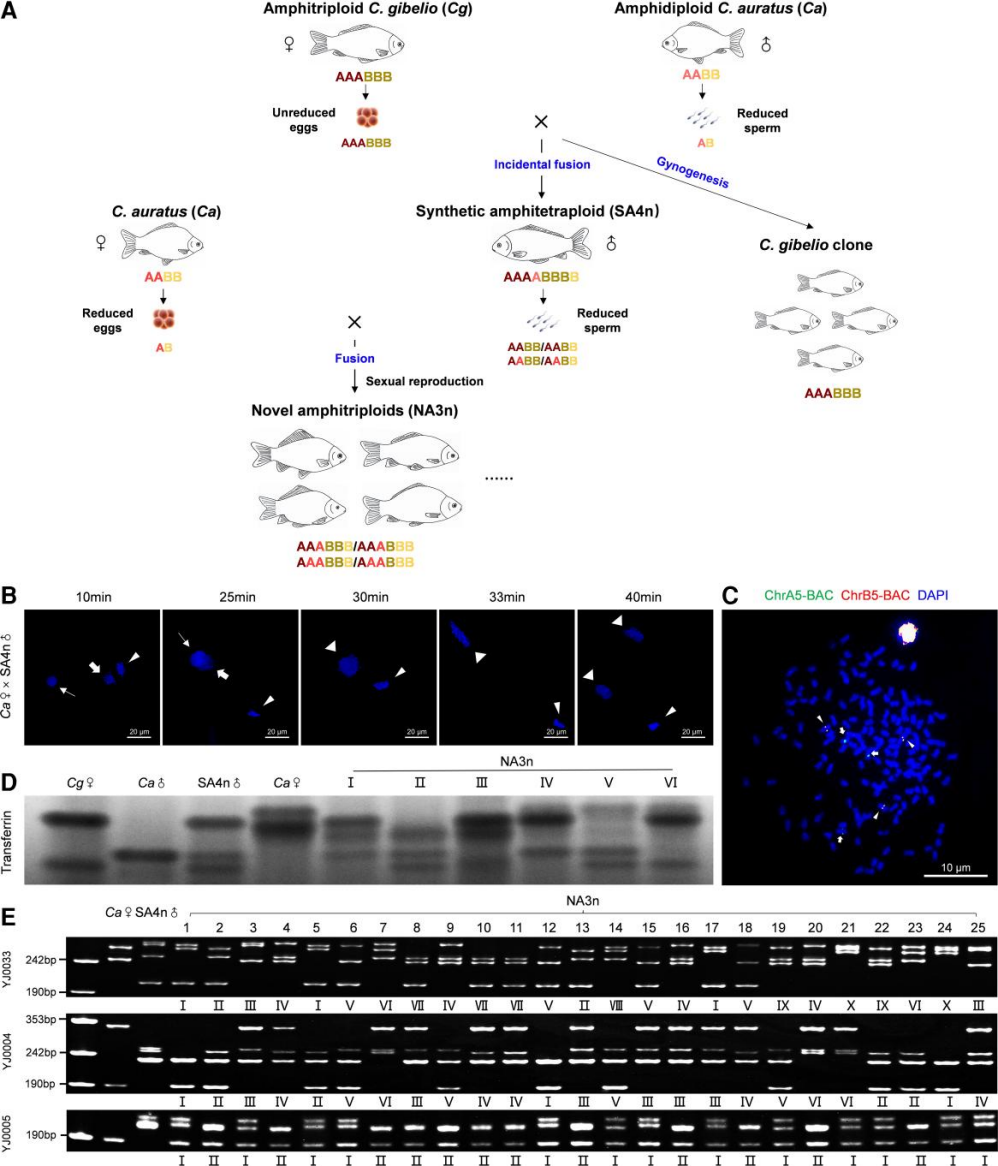
Generation and genetic diversity of the novel amphitriploids (NA3n). (*A*) Schematic diagram of the generation process. Dark red, bright red, dark yellow, and bright yellow represent the subgenomes A and B of *C. gibelio* and *C. auratus*, respectively. (*B*) Nuclear behaviors in the fertilized eggs of *Ca*♀ × SA4n♂. Sperm nucleus and female pronucleus are indicated by thin and thick arrows, respectively. Second polar body is indicated by thin triangular arrowheads, and the nuclei of zygotes are indicated by thick triangular arrowheads. (*C*) FISH analysis of *Cgdmrt1-A*-BAC-DNA and *Cgdmrt1-B*-BAC-DNA on metaphase chromosome A5 (Chr A5) and Chr B5, respectively. BAC-DNA was labeled with DIG (green) or biotin (red), respectively, and all metaphase chromosomes (blue) were counterstained with DAPI. (*D*) TF electrophoretic patterns of NA3n on 10% PAGE gel with their parents (*Ca*♀ and SA4n♂) and the parents of SA4n♂ (*Cg*♀ and *Ca*♂). (*E*) Microsatellite genotypes of NA3n with their parents amplified by the primers of YJ0033, YJ0004 and YJ0005. *Cg*, *C. gibelio*; *Ca*, *C. auratus*; NA3n, novel amphitriploid; SA4n, synthetic amphitetraploid; ♀, female; ♂, male; I–X, genotype 1–10.

As shown in figure 1*A*, novel amphitriploids were generated, and they were revealed to result from sexual reproduction as the sperm nucleus of synthetic amphitetraploids was observed to fuse with the female pronucleus of *C. auratus* during fertilization and early embryogenesis (fig. 1*B*). The adults were characterized as novel amphitriploids because the metaphases possessed ∼150 chromosomes and contained three homologous chromosomes with green signals from *dmrt1-A*-bacterial artificial chromosome (BAC)-DNA (chromosome A5, ChrA5) or *viperin-A*-BAC-DNA (ChrA17) of subgenome A, as well as three homologous chromosomes with red signals from *dmrt1-B*-BAC-DNA (ChrB5) or *viperin-B*-BAC-DNA (ChrB17) of subgenome B (fig. 1*C* and supplementary fig. S1, Supplementary Material online). Subsequently, we analyzed the transferrin (TF) electrophoretic patterns (Li and Gui 2008). As shown in figure 1*D*, the fertile amphitetraploid male inherits all TF bands from its maternal *C. gibelio* and paternal *C. auratus*. A total of six novel TF electrophoretic patterns were observed from 25 individuals of novel amphitriploids, in which each of them possessed two bands from the paternal amphitetraploid and one band from the maternal *C. auratus*. The novel amphitriploids were also subjected to microsatellite genotype analysis via 15 pairs of primers (Zhao et al. 2021). Diverse microsatellite genotypes were observed in the analyzed 25 individuals, and the number of alleles per locus ranged from 1 to 8 (fig. 1*E* and supplementary fig. S2, Supplementary Material online). For example, two and four alleles were detected by primer YJ0033 in maternal *C. auratus* and the paternal amphitetraploid, respectively, and all of the analyzed novel amphitriploids randomly inherited one allele from maternal *C. auratus* and two alleles from the paternal amphitetraploid. A total of 10 microsatellite genotypes were observed in the 25 analyzed individuals among the novel amphitriploids. Similarly, six (primer YJ0004) and two (primer YJ0005) microsatellite genotypes that randomly inherited some maternal and paternal alleles were observed among the 25 individuals (fig. 1*E*). These results indicate that the novel amphitriploids generated from sexual reproduction by backcrossing the amphitetraploid male with *C. auratus* females possess high genetic diversity.

### Diverse Chromosome Compositions and Genomic Changes of the Novel Amphitriploids

**Fig. 2.**
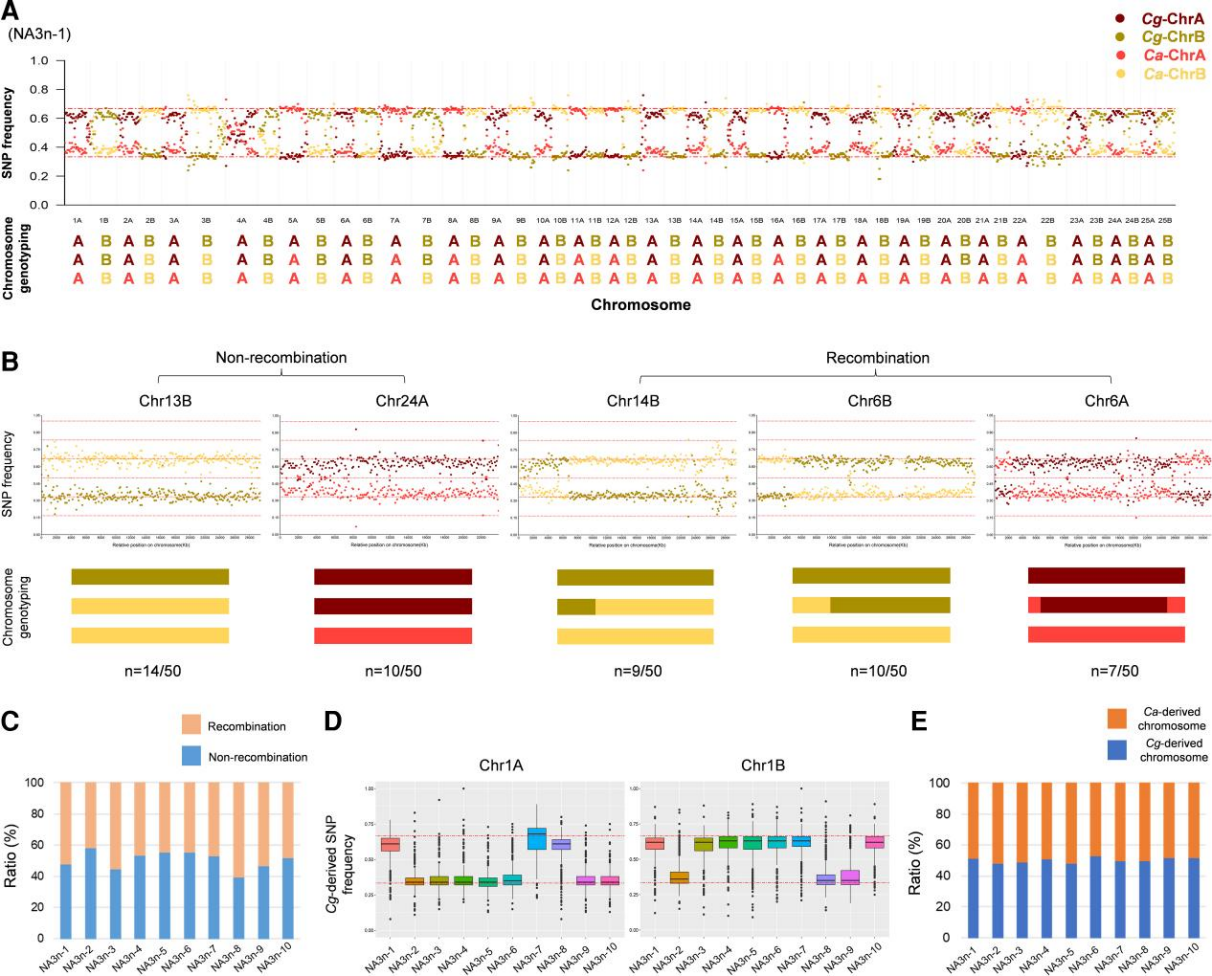
Chromosome composition analysis of the novel amphitriploids (NA3n). (*A*) Whole-genome genotyping data from a novel amphitriploid individual (NA3n-1). Each point represents the average SNP frequency in 1 Mb windows ordered across the assembled genome of *C. auratus* (Chr1*A* and Chr1*B* to Chr25*A* and Chr25*B*). Dark red, bright red, dark yellow, and bright yellow represent the genotypes of *C. gibelio* and *C. auratus* in subgenomes A and B, respectively. Genotyping of chr4*A* was conducted according to the SNP frequencies in the windows from the posterior region due to the significant differences in repeat sequences of the anterior region between *C. gibelio* and *C. auratus*. Genotyping for another nine individuals of NA3n are shown in supplementary figure S4, Supplementary Material online. (*B*) Genotyping of five representative chromosomes of NA3n-1. Each point represents the average SNP frequency in 100 kb windows ordered along the chromosome. Genotyping for another 45 chromosomes of NA3n-1 is shown in supplementary figure S5, Supplementary Material online. (*C*) Average ratio of nonrecombinant and recombinant homologous chromosomes groups in ten individuals of NA3n. (*D*) Box plot of *Cg*-derived SNP frequency statistics of Chr1*A* and Chr1*B* in ten individuals of NA3n. Box plot of *Cg*-derived SNP frequency statistics of another 48 chromosomes are shown in supplementary figure S6, Supplementary Material online. (*E*) Average ratio of *Cg*-derived and *Ca*-derived chromosomes in ten individuals of NA3n.

To uncover genomic changes, we conducted whole-genome resequencing of ten novel amphitriploid individuals, as well as the maternal *C. auratu*s ♀, and the parent *C. gibelio* ♀ and *C. auratu*s ♂ of the paternal amphitetraploid male, to analyze their chromosomal compositions and recombination as described in *Caenorhabditis* nematodes (Lamelza et al. 2019) (supplementary table S1, Supplementary Material online). A total of 844,982 effective single nucleotide polymorphisms (SNPs) that were homozygous and differed between *C. gibelio* and *C. auratus* (see Materials and Methods for details) were used to distinguish the genotypes of *C. gibelio* or *C. auratus* in the novel amphitriploids. Among the 844,982 SNPs, a total of 589,200–625,938 effective heterozygous SNPs were used to conduct chromosomal genotyping in ten novel amphitriploids by calculating the mean SNP frequencies in 1 Mb windows (supplementary fig. S3A, Supplementary Material online). An approximate SNP frequency of 0.33 (1/3) for *C. gibelio* or *C. auratus* was inferred for one chromosome inherited from *C. gibelio* or *C. auratus*, whereas an approximate SNP frequency of 0.67 (2/3) for *C. gibelio* or *C. auratus* was inferred for two chromosomes inherited from *C. gibelio* or *C. auratus*, respectively (supplementary fig. S3B, Supplementary Material online). Through the chromosome genotyping, we identified the chromosome compositions of ten novel amphitriploids. Although they inherited one whole chromosome set (AB) from *C. gibelio* and one whole chromosome set (AB) from *C. auratus* (fig. 2*A* and supplementary fig. S4, Supplementary Material online), the third chromosome set was randomly derived from subgenome A or subgenome B of *C. gibelio* or *C. auratus*, and thus the chromosome compositions were diverse and variable among the ten novel amphitriploids.

Subsequently, we analyzed the genotypes of all 50 homologous chromosome groups in one novel amphitriploid (NA3n-1) by calculating the mean SNP frequencies in 100 kb windows (supplementary fig. S5, Supplementary Material online) and revealed five categories of representative chromosome compositions. As shown in figure 2*B*, homologous chromosome groups, such as Chr13B and Chr24A, displayed continuous and consistent SNP frequencies along the whole chromosome, implying that no recombination event had occurred between homologs derived from *C. gibelio* and *C. auratus*. However, discontinuous and reverse SNP frequencies existed at one end of Chr14B and Chr6B, and two ends of Chr6A, implying that one or two recombination events had taken place in Chr14B, Chr6B, and Chr6A. Moreover, we determined all nonrecombination and recombination occurrences in another nine novel amphitriploids. The ratios of the nonrecombination and recombination chromosome groups were close to 1:1 in all ten individuals (fig. 2*C* and supplementary table S2, Supplementary Material online).

Finally, the mean *Cg*-derived SNP frequencies of each homologous chromosome group from ten novel amphitriploids were calculated. The genotyping data uncovered random chromosome compositions from *C. gibelio* and *C. auratus* in novel amphitriploids (fig. 2*D* and supplementary fig. S6, Supplementary Material online). Three novel amphitriploids (NA3n-1, NA3n-7, and NA3n-8) possessed two Chr1A chromosomes from *C. gibelio*, whereas the other seven individuals had two Chr1A chromosomes from *C. auratus.* The inheritance pattern of Chr1B among the ten novel amphitriploid individuals differed from that of Chr1A. Significantly, the average ratio of chromosomes inherited from *C. gibelio* or *C. auratus* was close to 1:1 in all ten novel amphitriploids (fig. 2*E* and supplementary table S3, Supplementary Material online).

Taken together, the above results reveal that diverse chromosome compositions and significant genomic changes should result mainly from homologous recombination between homologs derived from *C. gibelio* and *C. auratus*, as well as nonhomologous independent assortment, which may occur during meiosis in spermatogenesis of the amphitetraploid. These changes lead to high genomic diversity in novel amphitriploids.

### Novel Amphitriploid Females Recover Unisexual Gynogenesis Ability

**Fig. 3.**
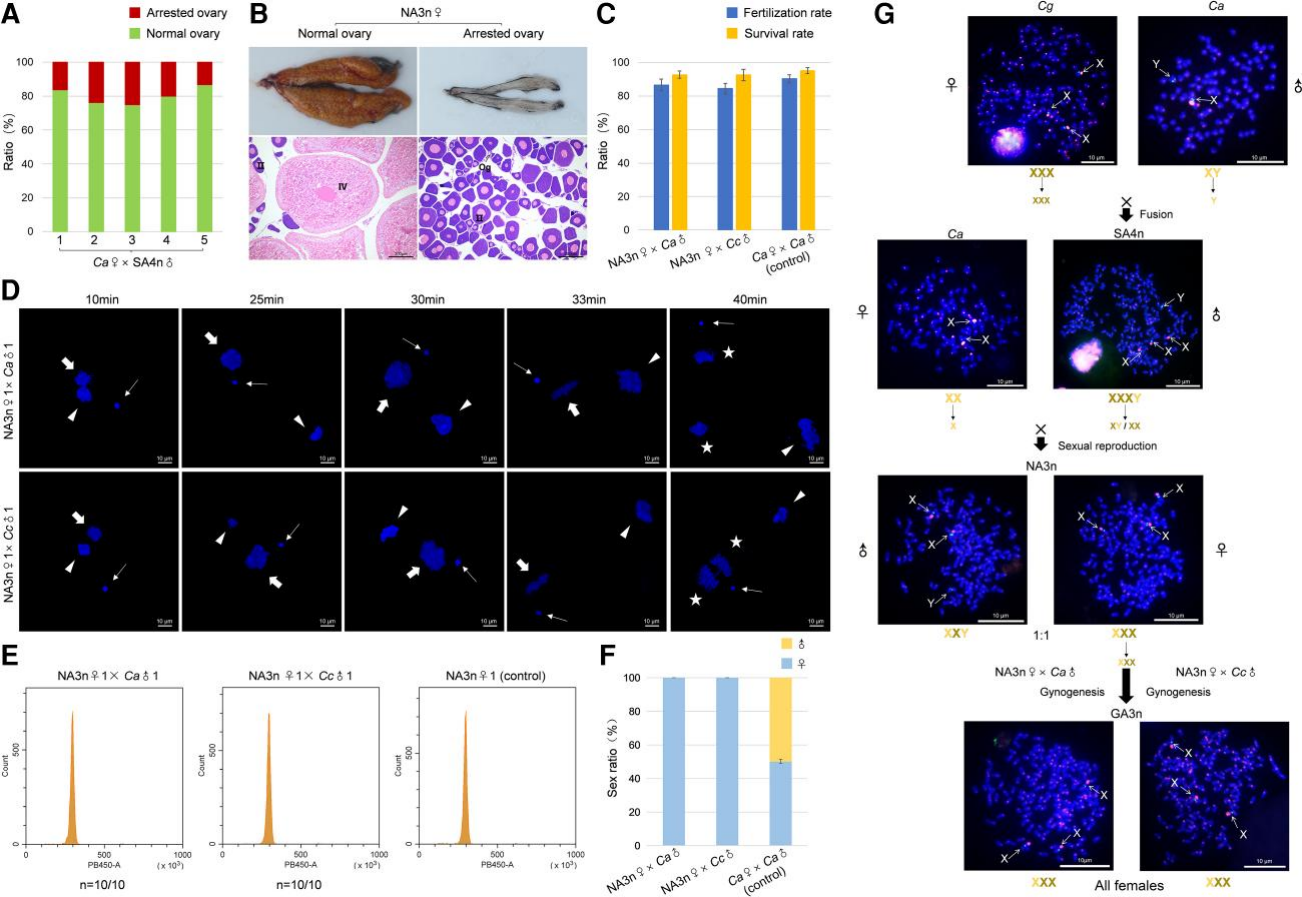
Fertility and sex chromosome inheritance of novel amphitriploid females (NA3n♀). (*A*) Ratio of normal and arrested ovary in NA3n♀ from five groups. (*B*) Histology structure of ovary in NA3n♀. Og, oogonia; II, growth stage oocyte; IV, maturing ovary. (*C*) Fertilization and survival rates of three different crossed combinations (NA3n♀ × *Ca*♂, NA3n♀ × *Cc*♂, and *Ca*♀ × *Ca*♂). (*D*) Nuclear behaviors in the fertilized eggs of one novel amphitriploid female (NA3n♀1) crossed with one *C. auratus* male (*Ca*♂1) or one *C. carpio* male (*Cc*♂1), respectively. Sperm nucleus and female pronucleus are indicated by thin and thick arrows, respectively. Second polar body is indicated by triangular arrowheads, and the nuclei of the zygotes after the first mitosis are indicated by asterisks. (*E*) Histograms of DNA content of blood cells from the offspring of two crossed combinations (NA3n♀1 × *Ca*♂1 and NA3n♀1 × *Cc*♂1) via flow cytometry. Blood cells from NA3n♀1 were used as the control. (*F*) Sex ratio of the offspring from three crossed combinations (NA3n♀ × *Ca*♂, NA3n♀ × *Cc*♂, and *Ca*♀ × *Ca*♂). G, Inheritance of sex chromosomes in NA3n and their offspring. FISH analysis of sex chromosomal specific-probe (green) and *C. auratus* genomic probe (red) were performed during the metaphase of the *C. gibelio* female, the *C. auratus* male and female, the SA4n male, the NA3n female and male, and the offspring from two crossed combinations (NA3n♀ × *Ca*♂ and NA3n♀ × *Cc*♂). X and Y chromosomes are indicated by arrows. X and Y chromosomes both contain green signals of sex chromosomal specific fragment. The short arms of the X chromosome are covered by highly intensive signals of *C. auratus* genomic sequences (Lu et al. 2021). NA3n, novel amphitriploid; SA4n, synthetic amphitetraploid; *Ca*, *C. auratus*; *Cc*, *C. carpio*; GA3n, gynogenetic amphitriploid; ♀, female; ♂, male.

To analyze the common reproductive characteristics, five groups of novel amphitriploids were generated by mating the amphitetraploid male with five *C. auratus* females. During the reproductive season, we examined the sex ratio and gonad development of the novel amphitriploids (supplementary table S4, Supplementary Material online). Among them, ∼80% of females developed normal ovaries with numerous maturing oocytes (IV), and these females could spawn mature eggs (fig. 3*A*and*B*). The remaining females had small and translucent ovaries with large numbers of growth stage oocytes (II) (fig. 3*A*and*B*andsupplementary table S5, Supplementary Material online), indicating that oocyte development was arrested at the primary growth stage.

To evaluate fertility, novel amphitriploid females were crossed with *C. auratus* males and heterogenous common carp (*Cyprinus carpio*) males (fig. 3*C* and supplementary table S6, Supplementary Material online). The average fertilization rate and larval survival rate of both crosses were similar to those of the control group (*C. auratus*♀ × *C. auratus*♂). It appears that novel amphitriploid females regain the gynogenesis ability, in which sperm is required as a physiological trigger, independent of species and ploidy differences. To confirm this speculation, we traced nuclear behaviors during the process of fertilization. As shown in figure 3*D*, the sperm nuclei of *C. auratus* and *C. carpio* undergo typical development behavior of gynogenesis after entering the egg, remaining in a condensed state and failing to fuse with the female pronucleus. The female pronucleus accomplishes all steps of the first mitosis without incorporating the sperm nucleus, which differs from sexual reproduction (fig. 1*B*) and meiotic hybridogenesis (Crespo-López et al. 2006). The offspring from both crosses had blood cell DNA content identical to that of their maternal NA3n♀ (fig. 3*E* and supplementary table S7, Supplementary Material online), and the offspring were all female (fig. 3*F* and supplementary table S8, Supplementary Material online). These results indicate that the novel amphitriploid females regained the unisexual gynogenesis ability. Using the recovered gynogenesis, diverse unisexual amphitriploid clones were reproduced (supplementary fig. S7, Supplementary Material online).

Consistent with our previous study (Lu et al. 2021), the fertile synthetic amphitetraploid male had three X chromosomes and one Y chromosome. When it was mated with a *C. auratus* female, the novel amphitriploid females inherited three X chromosomes, whereas the males obtained two X chromosomes and one Y chromosome. The novel amphitriploids with three X chromosomes reproduced via gynogenesis, and further generated gynogenetic amphitriploids with three X chromosomes (fig. 3*G*).

### Revelation of Two Types of Primary Oocyte in Novel Amphitriploids

**Fig. 4.**
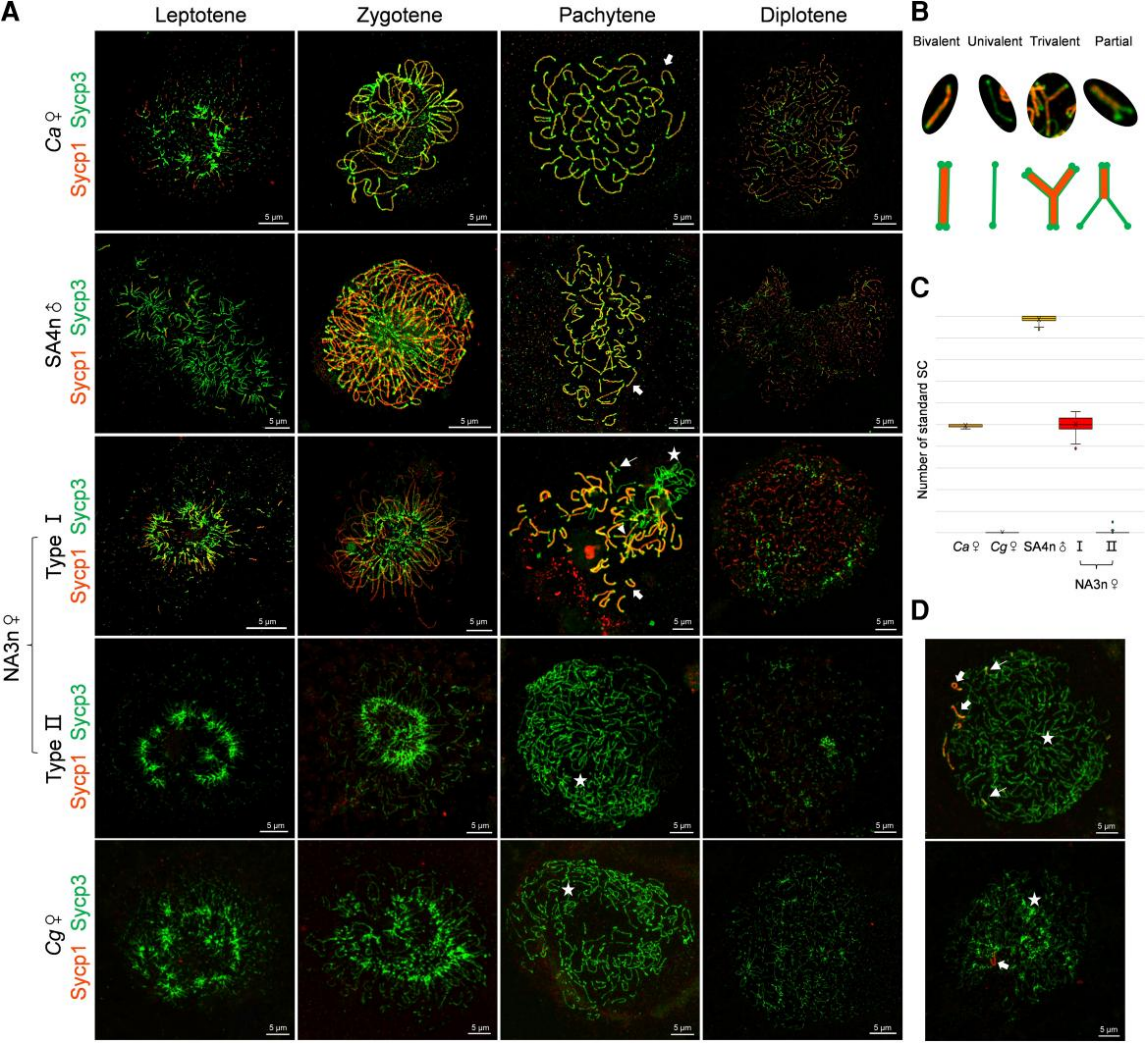
SC formation in primary oocytes or spermatocytes of novel amphitriploid females (NA3n♀) and their parents. (*A*) Chromosomal spreads of primary oocytes or spermatocytes were coimmunostained by anti-Sycp1 (red) and anti-Sycp3 (green) antibodies. Primary oocytes or spermatocytes were staged according to the staining patterns of Sycp1 and Sycp3. At the pachytene stage, thick arrows, thin arrows, triangular arrowheads, and asterisks indicate standard bivalent, partial paired bivalent, trivalent, and univalent SC respectively. Females of *Ca* (*n* = 3), *Cg* (*n* = 3), and NA3n (*n* = 10) at 90 dph were sampled for oocyte chromosomal spreads, and the SA4n male at 420 dph was sampled for spermatocyte chromosomal spreads. (*B*) Schematic mode of four representative SCs in type I oocytes of NA3n♀. (*C*) Statistics of standard SCs from the five kinds of primary oocyte or spermatocyte (*n* = 50). (*D*) Special cases showing sporadic SC assembly in type II oocytes of NA3n♀. SC, synaptonemal complex; *Cg*, *C. gibelio*; *Ca*, *C. auratus*; SA4n, synthetic amphitetraploid; NA3n, novel amphitriploid; ♀, female; ♂, male.

One disadvantage of triploidy is the potential difficulty of homolog synapsis during normal meiosis (Comai 2005). To reveal the cytological mechanism for how the novel amphitriploid females could bypass this barrier, we coimmunostained nuclear microspreads of primary oocytes or spermatocytes with antibodies for synaptonemal complex (SC) transverse element (Sycp1) and lateral element (Sycp3) (Page and Hawley 2004) to investigate the dynamic processes of SC formation during the corresponding meiotic prophase I (fig. 4*A*). The typical dynamic processes of SC formation were shown in the sexual *C. auratus* female (fig. 4*A*, *Ca*♀). At the late leptotene stage, Sycp3-positive threads began to form bouquet foci and the short stretches of Sycp1 appeared close to the Sycp3 lines. Along the progression of the zygotene stage, Sycp1 loaded and extended along Sycp3 axial signals, and a typical bouquet was formed. At the pachytene stage, 50 SCs were completely assembled, and these began to degrade at the diplotene stage (fig. 4*A*, *Ca*♀). The amphitetraploid male also displayed typical processes of SC formation in the spermatocytes, in which about 100 bivalents appeared in most spermatocytes (*n* = 43/50) at the pachytene stage (fig. 4*A*, SA4n♂), and a few of the univalents stained only the Sycp3 signal and one tetravalent were also observed in six spermatocytes and one spermatocyte, respectively (supplementary fig. S8, Supplementary Material online). Unexpectedly, two distinct types of primary oocyte microspreads were observed in the same novel amphitriploid female, characterized by the presence or absence of Sycp1 signals (fig. 4*A*, NA3n♀). In type I oocytes, similar bouquet foci and SC formation were seen at the leptotene, zygotene, and pachytene stages, but only ∼50 SC bivalents with Sycp3 and Sycp1 signals were assembled, and other unpaired univalents kept only the Sycp3 signal and twisted around each other (fig. 4*A*, NA3n♀, type I). Occasionally, SC trivalents with partial side-by-side synapsis among three homologous chromosomes and partial paired SC bivalents were observed at the pachytene stage (fig. 4*B*). In type II oocytes, only the Sycp3 green signal was stained, whereas the Sycp1 protein signal was absent, and the unpaired univalents twisted around each other (fig. 4*A*, NA3n♀, type II), indicating that SC assembly was not fulfilled within the oocytes. Significantly, the chromosomal behavior in type II oocytes was identical to that observed in clone A^+^ (fig. 4*A*, *Cg*♀) and in a wild clone (clone H) of *C. gibelio* (Gao et al. 2017) (supplementary fig. S9, Supplementary Material online). Figure 4*C* shows the counting SC number from the five types of primary oocyte or spermatocyte. Occasionally, one to five assembled SCs were observed in some type II oocytes (fig. 4*D*).

### Distinct Development Fates of Types I and II Oocytes

**Fig. 5.**
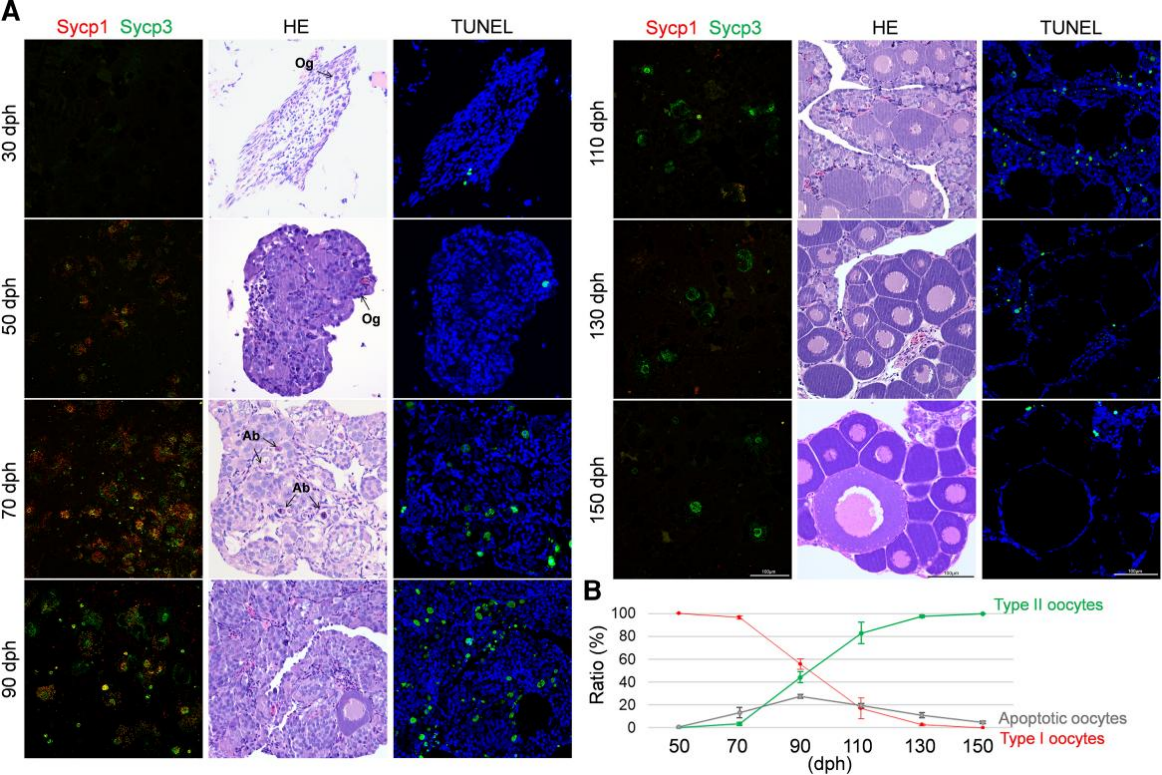
Apoptotic feature in primary oocytes during ovarian maturation of the novel amphitriploid female (NA3n♀). (*A*) SC spreads of primary oocytes, histological characteristics, and apoptotic feature during ovarian maturation of NA3n. SC spreads of primary oocytes were coimmunostained by anti-Sycp1 (red) and anti-Sycp3 (green) antibodies. Histological characteristics of corresponding ovaries were stained with HE and apoptotic signals were detected by TUNEL. Nuclei were stained with DAPI (blue). (*B*) Proportion of two types of primary oocyte and ratio of apoptotic oocytes during ovarian maturation of NA3n. According to the numbers of type I and type II oocytes from three microscopic vision fields per NA3n individual (*n* = 3) at different stages, the mean proportions of two types of primary oocyte were calculated. The mean ratio of apoptotic oocytes was calculated according to the number of oocytes with apoptotic signals to total the number of oocytes in three microscopic vision fields per NA3n individual (*n* = 3). Ab, apoptotic body; dph, days posthatching; Og, oogonia.

To understand the occurrence time and ratio of the two types of oocyte, we first investigated the microspreads of primary oocytes within ovaries at different developmental stages in novel amphitriploids using Sycp1 and Sycp3 immunostaining (as described above). At 30 days posthatching (dph), a few oogonia (Og) were scattered in the gonadal primordia and signals of Sycp1 and Sycp3 were barely detected (fig. 5*A*). Og massively proliferated in cysts from 50 dph, and some began to differentiate into primary oocytes. Nearly all of the oocyte microspreads belonged to type I oocytes, simultaneously showing intense Sycp1- and Sycp3-positive signals. Abundant primary oocytes were detected at 70 dph, indicating that large numbers of Og had entered the meiotic prophase. At that time, the majority (96.6 ± 1.4%) of the microspreads still belonged to type I oocytes. At 90 dph, primary oocytes began to grow and differentiate. Interestingly, the number of type I primary oocytes progressively decreased, and about half (44.3 ± 4.6%) of the microspreads began to show type II oocyte characteristics, with only Sycp3-positive signals. Along with ovary development (110–150 dph), type I primary oocytes quickly declined, and type II primary oocytes gradually increased (fig. 5*A*).

Compared with *C. gibelio* and *C. auratus* (Gan et al. 2021), the ovary development of the novel amphitriploids was slower and arrested at the oogonium proliferation and primary oocyte differentiation stage for 1–2 months. Therefore, we speculated that during the arrested period from 70 to 110 dph (fig. 5*A*), type I and type II primary oocytes may experience distinct development fates. To verify this, we performed TUNEL staining and haematoxylin–eosin (HE) staining analyses of the corresponding ovaries from 30 to 150 dph. As shown in figure 5, primary oocyte apoptosis should be the key to the distinct development fates. In the 50 dph ovaries, only a small number (0.7 ± 0.1%) of sporadic TUNEL signals were detected. The number of apoptotic oocytes greatly increased at 70 dph and reached its peak (27.7 ± 1.9%) at 90 dph (fig. 5*B*). HE staining showed that the apoptotic signals were mainly merged with primary oocytes in cysts. The primary oocytes had apoptotic hallmarks, such as nuclear swelling and fragmentation, apoptotic body formation, and even vacuolization (fig. 5*A*). Along with ovary development and oocyte growth (fig. 5*A*, 130 and 150 dph), the apoptotic signals became diminished. Figure 5*B* shows the statistical data of three repeated observations, in which the distinct development fates of types I and II primary oocytes occurred at the arrested period from about 70 to 110 dph. At this stage, type I primary oocytes degenerated through cell apoptosis, and only type II primary oocytes continued to develop into mature oocytes and eggs.

### Meiotic Failure of Type I Oocytes and Alternative Ameiotic Pathway of Type II Oocytes

**Fig. 6.**
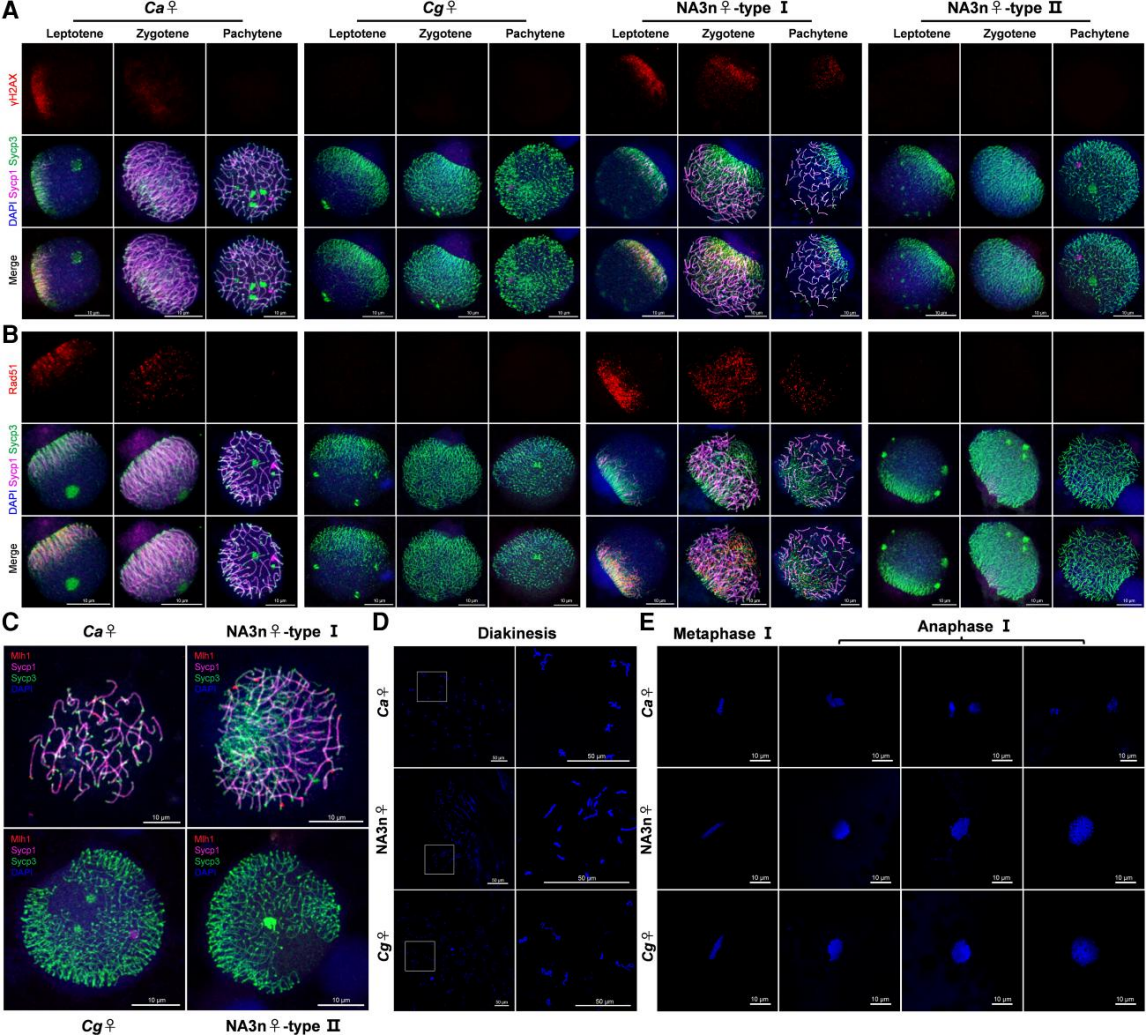
Chromosome behaviors of two types of oocyte from the novel amphitriploids (NA3n). (*A* and *B*) Double-strand break (DSB) formation and repair marked by γH2AX (*A*) and Rad51 (*B*) in primary oocytes of sexual *C. auratus*, unisexual *C. gibelio* and the novel amphitriploids. Chromosomal spreads of primary oocytes were coimmunostained by anti-γH2AX (*A*) (red) or anti-Rad51 (*B*) (red) with anti-Sycp1 (magenta) and Sycp3 (green) antibodies. Nuclei were stained with DAPI (blue). Primary oocytes were staged according to the staining patterns of Sycp1 and Sycp3. Females of *Ca* (*n* = 3), *Cg* (*n* = 3), and NA3n (*n* = 10) at 90 dph were sampled for oocyte chromosomal spreads. (*C*) Recombination sites were identified by anti-Mlh1 antibody (red) at the pachytene stage. SCs were visualized by anti-Sycp1 (magenta) and anti-Sycp3 (green) antibodies. Nuclei were stained with DAPI (blue). (*D*) DAPI-stained chromosome spread of GV at diakinesis. Fifty eggs from *Ca* (*n* = 3), *Cg* (*n* = 3), and NA3n (*n* = 5) were collected to isolate GVs. (*E*) Nuclear behaviors at metaphase I and anaphase I stage. Fifty eggs from *Ca* (*n* = 3), *Cg* (*n* = 3), and NA3n (*n* = 5) were collected for cytological observation. *Cg*, *C. gibelio*; *Ca*, *C. auratus*; NA3n, novel amphitriploid; ♀, female.

To investigate the cytological mechanisms underlying the distinct developmental fates of two types of primary oocyte, we used immunofluorescence staining to examine the dynamic changes of γH2AX and Rad51, as well as Sycp1 and Sycp3, in the spread nuclei of oocytes from sexual *C. auratus*, gynogenetic *C. gibelio*, and the novel amphitriploids. γH2AX is a phosphorylated form of the histone variant, and a marker for DNA double-strand breaks (DSBs) (Rahmanian and Shokrzadeh 2021); Rad51 is a DNA recombinase that promotes the recombinational repair of DSBs (Takemoto et al. 2020). In *C. auratus*, abundant γH2AX and Rad51 signals were observed at leptotene, accompanied by the assembling of Sycp1 and Sycp3 at one pole of the nucleus. These signals gradually decreased at zygotene and diminished at pachytene (fig. 6*A*and*B*, *Ca*♀). However, the γH2AX signal was barely detectable in oocytes of *C. gibelio* (fig. 6*A*, *Cg*♀ and supplementary fig. S9A, Supplementary Material online), and the Rad51 signal was absent in 90% and 86% of oocytes (*n* = 50) of clone A^+^ (fig. 6*B*, *Cg*♀) and wild clone H (supplementary fig. S9B, Supplementary Material online) of *C. gibelio*, respectively. Sporadic Rad51 signals were detected in the remaining analyzed oocytes (supplementary fig. S10, Supplementary Material online). The results implied that both DSB formation and homologous recombination were largely inhibited within the oocytes of *C. gibelio.* Consistent with the presence or absence of Sycp1 loading to SCs, two types of oocyte from the novel amphitriploids also showed two distinct signals of γH2AX and Rad51. Type I oocytes showed abundant bright γH2AX and Rad51 signals at leptotene and decreased at zygotene, resembling the pattern seen in *C. auratus*. However, considerable γH2AX signals were still distributed mostly in subnuclear regions, where Sycp3 univalents clustered, and many Rad51 foci remained on the Sycp3-stained axes of univalents at pachytene (fig. 6*A*and*B*, NA3n♀—type I). The kinetics of the γH2AX and Rad51 signals suggest that DSB repair may be incomplete in type I oocytes, and these oocytes may trend toward apoptosis due to the difficulty in accomplishing meiosis. By contrast, the cytological behaviors in type II oocytes (fig. 6*A*and*B*, NA3n♀—type II) were identical to those observed in *C. gibelio* (fig. 6*A*and*B*, *Cg*♀ and supplementary fig. S9A and B, Supplementary Material online), implying that they adopt the same ameiotic pathway as *C. gibelio* to bypass the meiosis bottleneck.

Crossover (CO) formation is the key to ensuring correct pairing and separation of homologs (Xin et al. 2021). The recombination sites at the pachytene stage were identified by an antibody against Mlh1, a component of the DNA mismatch repair protein complex participating in the formation of COs (Cannavo et al. 2020). One to two Mlh1 foci were localized to each assembled SC bivalent of *C. auratus* (fig. 6*C*, *Ca*♀). As expected, type I oocytes of the novel amphitriploids had one to two Mlh1 foci per SC bivalent, whereas little Mlh1 signal was detected in their unpaired univalents (fig. 6*C*, NA3n♀—type I). The CO Mlh1 signal was absent in *C. gibelio* (fig. 6*C*, *Cg*♀ and supplementary fig. S9C, Supplementary Material online). Similarly, no CO Mlh1 signal was detected in 98% (*n* = 50) of the type II oocytes (fig. 6*C*, NA3n♀—type II), except for one Mlh1 signal that was observed in one type II oocyte (supplementary fig. S11, Supplementary Material online).

Subsequently, we conducted 4′,6-diamidino-2-phenylindole (DAPI) staining to examine the homologous chromosome pairing in germinal vesicles (GVs) at diakinesis (fig. 6*D*). Approximately 50 standard bivalents with chiasmata appeared in the GV of *C. auratus* at diakinesis (fig. 6*D*, *Ca*♀), whereas only univalent chromosomes existed in the corresponding GV of the novel amphitriploid (fig. 6*D*, NA3n♀), again, identical to that in observed in *C. gibelio* (fig. 6*D*, *Cg*♀ and supplementary fig. S9D, Supplementary Material online). This also confirmed that only type II oocytes develop into mature eggs.

In addition, we observed the nuclear events at the metaphase and anaphase stages of the corresponding meiosis I process. As expected, homolog segregation and first polar body exclusion could be seen in *C. auratus* (fig. 6*E*, *Ca*♀), but these nuclear events were not observed throughout the process of oocyte maturation in the novel amphitriploids (fig. 6*E*, NA3n♀) or in *C. gibelio* (fig. 6*E*, *Cg*♀ and supplementary fig. S9E, Supplementary Material online), implying that the novel amphitriploids adopted the identical ameiotic pathway used by *C. gibelio* to produce unreduced eggs.

### Unisexual Gynogenesis Can be Inherited Stably and Generates Diverse Clones

**Fig. 7.**
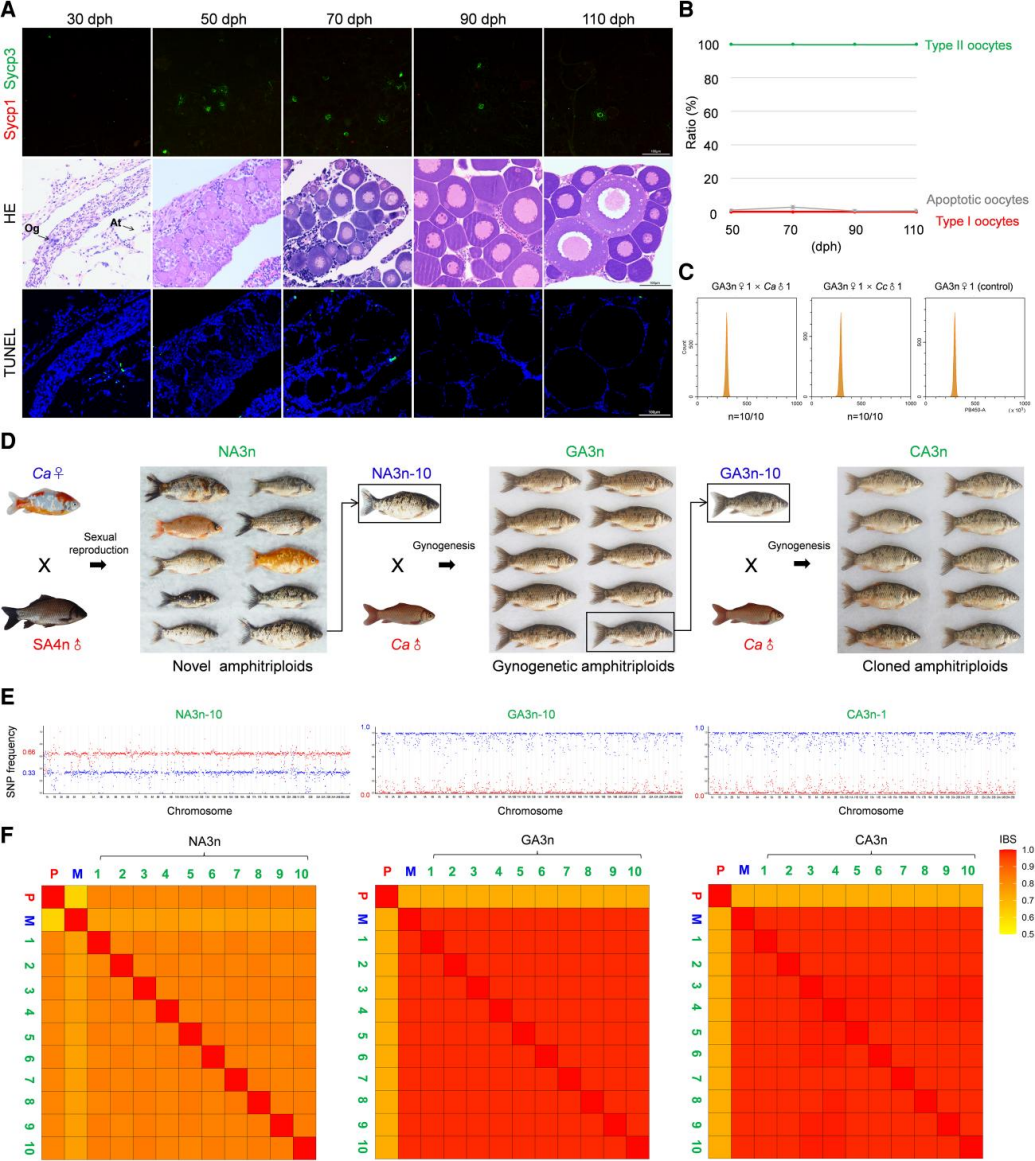
Stabilized gynogenesis ability and high genetic similarity in the offspring of the novel amphitriploid (NA3n). (*A*) SC spreads of primary oocytes, histological characteristics, and apoptotic feature during ovarian maturation of the gynogenetic amphitriploid females (GA3n). SC spreads of primary oocytes were coimmunostained by anti-Sycp1 (red) and anti-Sycp3 (green) antibodies. Histological characteristics of corresponding ovaries were stained with HE and apoptotic signals were detected by TUNEL. Nuclei were stained with DAPI (blue). (*B*) Proportion of two types of primary oocyte and ratio of apoptotic oocytes during ovarian maturation of GA3n. According to the numbers of type I and type II oocytes from three microscopic vision fields per GA3n individual (*n* = 3) at different stages, the mean proportions of two types of primary oocytes were calculated. The mean ratio of apoptotic oocytes was calculated according to the number of oocytes with apoptotic signal to total number of oocytes in three microscopic vision fields per GA3n individual (*n* = 3). (*C*) Histograms of DNA content of blood cells from the offspring of two crossed combinations (GA3n♀1 × *Ca*♂1 and GA3n♀1 × *Cc*♂1) via flow cytometry. Blood cells from GA3n♀1 were used as the control. (*D*) Breeding process and morphology of the novel amphitriploids (NA3n) and their two successive gynogenetic offspring (GA3n and CA3n). (*E*) Compositional analysis of parental chromosome sets in one individual from each of three amphitriploid generations by genome resequencing. Each point represents the average SNP frequency in 1 Mb windows ordered across the assembled genome of *C. auratus*. Blue and red represent the genotypes of maternal and paternal parent. (*F*) Heatmap from IBS analysis illustrating genetic diversity among ten offspring with their parents in three generations. At, adipose tissue; *Ca*, *C. auratus*; dph, days posthatching; SA4n, synthetic amphitetraploid; NA3n, novel amphitriploid; GA3n, gynogenetic amphitriploid; CA3n, cloned amphitriploid; Og, oogonia; *P*, paternal individual; M, maternal individual; ♀, female; ♂, male.

To determine the inheritance of reproductive characteristics, we further investigated the ovary development in the gynogenetic offspring of the novel amphitriploids. The ovaries developed faster in the gynogenetic offspring than in the novel amphitriploids because many primary oocytes entered into the growth stage at 50 dph, and yolk granules began to accumulate at 110 dph (fig. 7*A*), similar to *C. gibelio* and *C. auratus* (Gan et al. 2021; Wang, Li, Ding et al. 2022). Throughout oogenesis, only one type (type II) of primary oocyte was observed, and almost no (1.3 ± 0.9%) apoptotic signal was detected in the gynogenetic amphitriploids (fig. 7*A*and*B*). Moreover, the gynogenetic amphitriploids were crossed with *C. auratus* and *C. carpio* males, and the offspring had the same ploidy level as the maternal parent (fig. 7*C*). These results indicate that unisexual gynogenesis can be inherited stably in the gynogenetic offspring of novel amphitriploids.

To directly display the phenotypic differences, a female of a transparent and colorful *C. auratus* variant was chosen as the maternal parent to mate with the fertile synthetic amphitetraploid male. The resulting novel amphitriploids (NA3n) that originated from sexual reproduction showed obvious morphological differences each other, including body color, pattern of transparent scales, body shape, and size. Next, we randomly selected an individual (NA3n-10) to perform two successive unisexual gynogenesis to result in gynogenetic amphitriploids (GA3n) and cloned amphitriploids (CA3n). These offspring resembled their maternal parents, and no significant phenotypic differences were observed among individuals (fig. 7*D*).

Furthermore, a total of ten random individuals were selected from the novel amphitriploids (NA3n), gynogenetic amphitriploids (GA3n), and cloned amphitriploids (CA3n) to perform whole-genome resequencing and chromosome genotyping (supplementary table S1, Supplementary Material online). To distinguish the parental chromosome compositions of each generation, a total of 1,283,655 (NA3n), 513,536 (GA3n), and 543,284 (CA3n) effective SNPs were called for genotyping. Relative to their parents (*Ca*♀ and SA4n♂), the ten individuals of NA3n all exhibited ∼0.33 (1/3) SNP frequency of the maternal genotype and ∼0.66 (2/3) SNP frequency of the paternal genotype, inferring that NA3n individuals inherited one whole chromosome set from the maternal parent (*Ca*♀) and two whole chromosome sets from the paternal parent (SA4n♂) (fig. 7*E*, left and supplementary fig. S12, Supplementary Material online). Consistent with cytological observations (fig. 3*D*), the SNP frequencies of the maternal genotype in the ten individuals of GA3n (fig. 7*E*, middle) and CA3n (fig. 7*E*, right) approached 1.0 (3/3), whereas the SNP frequencies of the paternal genotype were almost 0.0 (0/3) (supplementary figs. S13 and S14, Supplementary Material online), indicating that GA3n and CA3n both inherited three whole chromosome sets from their maternal parents. Therefore, the genome resequencing and chromosome genotyping results confirmed that the novel amphitriploids not only recovered the unisexual gynogenesis ability, but also inherited stably.

To compare the genetic diversity among the three generations, we performed identity-by-state (IBS) analysis based on the SNP data (fig. 7*F*). The average genetic identity between two gynogenetic individuals of GA3n (0.983 ± 0.001) or CA3n (0.984 ± 0.001) was higher than that between two sexual individuals of NA3n (0.835 ± 0.008). The gynogenetic offspring of GA3n and CA3n all possessed very high genetic identity (>0.981) to their maternal parents but only about 0.720 to their paternal parents. The above data indicate that abundant genetic variations are produced among novel amphitriploids because they result from sexual reproduction between a fertile synthetic amphitetraploid male and a sexual amphidiploid *C. auratus* female. In addition, the novel amphitriploids recovered the unisexual gynogenesis ability and the ability was able to be inherited and to generate diverse clones. These clones were variable between clones and consistent among individuals within the same clone.

## Discussion

**Fig. 8.**
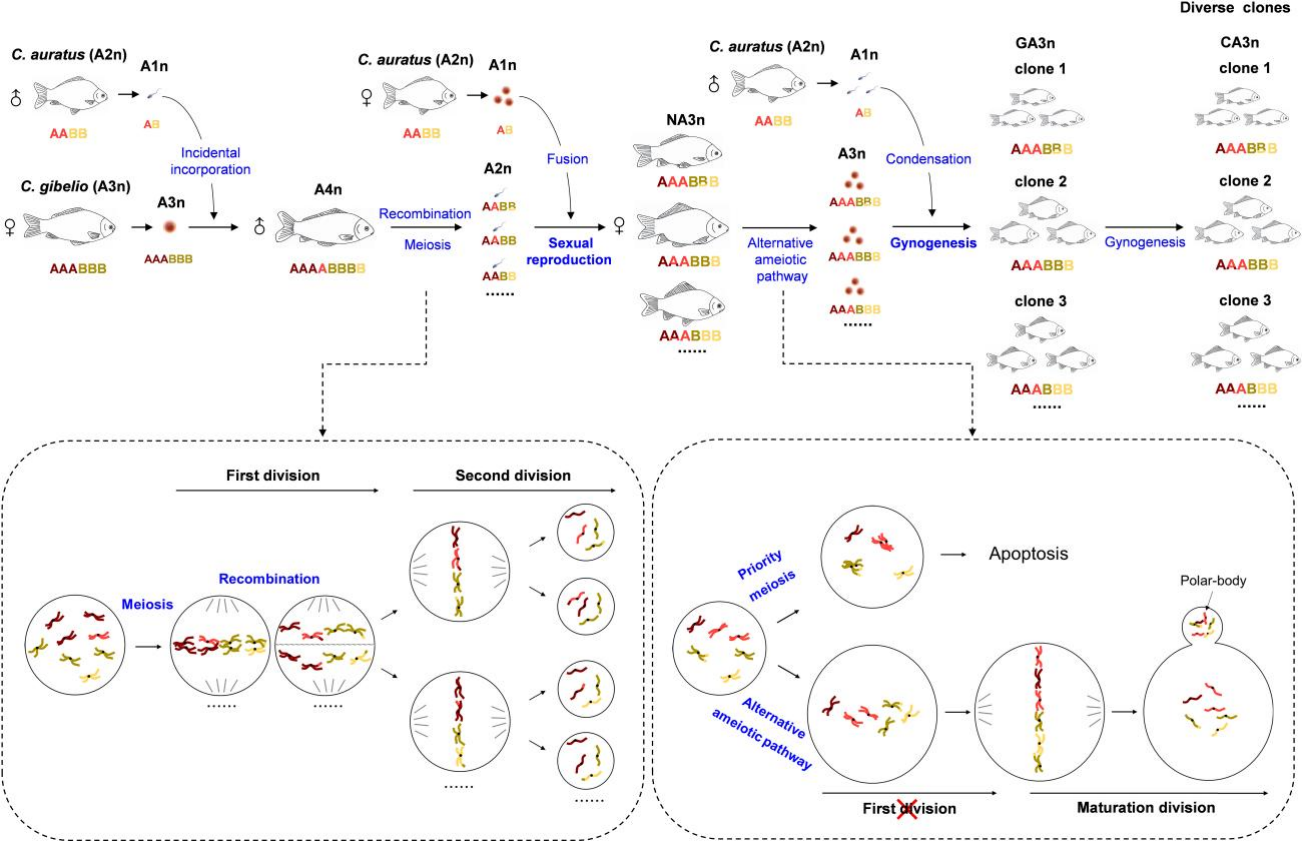
Schematic diagram for sexual reproduction (meiosis) and unisexual gynogenesis (ameiotic pathway) transition and clonal diversity driven via ploidy changes in *Carassius* complex. NA3n, novel amphitriploid; GA3n, gynogenetic amphitriploid; CA3n, cloned amphitriploid; ♀, female; ♂, male.

Unisexual and sexual reproduction transition, which facilitates an increase in genetic diversity, has already been proposed in the polyploid *Carassius* species complex (Lu et al. 2021; Mishina et al. 2021), and the complicated ecological and evolutionary impacts via the influence on the gene pool, diversification rate, and spatial distribution have also been suggested in some sexual and unisexual polyploid complexes (Janko et al. 2021). In this study, we provide comprehensive evidence that changes in ploidy, including from amphitriploid to amphitetraploid, then from amphitetraploid to novel amphitriploid, drive transition from unisexual to sexual reproduction and from sexual to unisexual reproduction, thereby leading to genomic and clonal diversity in the polyploid *Carassius* species complex. As shown in figure 8, unreduced amphitriploid eggs (A3n, AAABBB) of gynogenetic *C. gibelio* occasionally integrate amphihaploid (A1n, AB) sperm from sexual *C. auratus*, which result in synthetic amphitetraploids (A4n, AAAABBBB) (Lu et al. 2021). The genome addition from *C. auratus* prompts some amphitetraploid males to complete meiosis and recombination, and thereby to complete spermatogenesis and spawn amphidiploid (A2n, AABB) sperm. When the amphidiploid sperm (A2n, AABB) are backcrossed with amphihaploid eggs (A1n, AB) of *C. auratus*, a group of novel amphitriploids (NA3n, AAABBB) with divergent genomes are produced via sexual reproduction. Our results unequivocally show the occurrence of chromosomal recombination between *C. auratus* and *C. gibelio* homologs, and the segregation of chromosomes follows Mendel’ laws of segregation and independent assortment in the predicted ratio during sexual spermatogenesis of the fertile amphitetraploid, resulting in high genomic and clonal diversity in novel amphitriploids. Significantly, two distinct types of primary oocyte exist in novel amphitriploids, but only type II oocytes bypass the meiosis obstacle via an alternative ameiotic pathway to fulfill maturation division and recover the gynogenesis ability. The unreduced amphitriploid (A3n, AAABBB) eggs are inseminated with sperm from *C. auratus* or other fish species to perform two successive unisexual gynogenesis, and a new array of diverse gynogenetic amphitriploids (GA3n, AAABBB), as well as further cloned amphitriploids (CA3n) with abundant genetic diversity are produced. In previous studies, similar phenomena of reproduction transition and the impacts on genetic diversity of sexual–unisexual or asexual species complexes have also been discovered in dandelions (*Taraxacum*) (Van Dijk et al. 2003, 2016; Verduijn et al. 2004), planarian flatworm (*Schmidtea polychroa*) (D’Souza et al. 2004), and *S. alburnoides* (Alves et al. 2001; Crespo-López et al. 2006; Morgado-Santos et al. 2016), as well as in green toads (*Bufo viridis*) (Stöck et al. 2010, 2012). Therefore, unisexual and sexual reproduction transition is an important evolution strategy through which unisexual species maintain their clonal diversity. During reproduction dynamics, tetraploid or amphitetraploid males act as a bridge to generate new unisexual lineages and to facilitate genetic exchange from the sexual gene pool into unisexual lineages because they return to the meiotic situation rather than clonally reproducing. Significantly, recombination occurs between homologs from sexual species and unisexual species (interspecies recombination) during spermatogenesis, which has a considerable impact on the gene pools of these sexual–unisexual species complexes.

It remains unclear which molecular mechanisms trigger the reproduction transition switch. Most unisexual lineages are believed to be of hybrid and/or polyploid origin, implying a link between unisexual reproduction and hybridization/polyploidization (Vrijenhoek 1989; Neaves and Baumann 2011). Moritz et al. (1989) proposed that unisexuality arises when the genome divergences between parents fall within the right interval window: divergent enough to produce unreduced gametes by disrupting meiosis, yet not so divergent to maintain hybrid viability and fertility. The resurrection of new clones by hybridizing sexual parents in spined loaches (*Cobitis* sp.) indicates that unisexuality may be triggered by interspecific hybridization (Choleva et al. 2012) and may be associated with the phylogenetic divergence of parental species (Janko et al. 2018). Alternatively, apomixis in allodiploid *Boechera* hybrids might be due to the asynchronous expression of duplicate genes (Carman 1997), and the aposporous allodiploids might facilitate reversion from apomixis to sex (Carman et al. 2019). However, the switch between sexuality and unisexuality occurs from one generation to another in the polyploid *Carassius* complex, suggesting that it might be based on genetic machinery present in the direct ancestors of the clones. Recently, Hojsgaard and Schartl (2021) proposed that a nonrecombinant genetic assemblage might be an essential condition for the occurrence of a unisexual lineage. In this study, we observed the suppression of synapsis and the first meiotic division during oogenesis of *C. gibelio* clone A^+^ and wild clone H (figs. 4 and 6 and supplementary fig. S9, Supplementary Material online), being similar to clone F (Wang, Li, Xu et al. 2022). The results suggest that both wild and synthetic clones of *C. gibelio* might adopt the same ameiotic pathway to produce unreduced eggs. In comparison with the *C. auratus* genome, the *C. gibelio* genome shows intensive expansion and alterations in meiotic cell cycle-related genes and an oocyte-specific histone variant. These genomic alterations might be associated with the reproductive module of *C. gibelio* gynogenesis (Wang, Li, Xu et al. 2022). Although genetic factors or epigenetic states of genes in the genetic assemblage were not identified in this study, our data clearly suggest that novel amphitriploids might inherit the ameiotic oogenesis mechanism from *C. gibelio*, implying the reproductive module in *C. gibelio* has passed on to the novel amphitriploids.

Another intriguing finding in this study was to uncover two types of primary oocyte (with and without SC assembly) in the novel amphitriploids (figs. 4–6). One of the major challenges for triploids is their difficulty during meiosis, as the pairing and equal segregation of three homologous chromosomes is impossible (Comai 2005). Chromosome pairing failure often results in meiotic arrest at pachytene–metaphase I, followed by apoptosis, or aneuploid gamete production (Kuroda et al. 2019; Zhang et al. 2021). However, unisexual vertebrates develop unique mechanisms, such as oogonial fusion or premeiotic endoreplication in parthenogenetic lizards (*Aspidoscelis tesselata*) (Lutes et al. 2010) and *Cobitis* triploid females (Dedukh et al. 2020), as well as suppression of homolog synapsis and the first meiotic division in *P. formosa* (Schultz 1979), to bypass these barriers in meiosis. The current data indicate that both natural and novel amphitriploids in gynogenetic *C. gibelio* adopt an alternative ameiotic pathway, similar to that observed in *P. formosa* (Schultz 1979), via the suppression of DSB formation, homolog synapsis, and recombination, as well as the first meiotic division (figs. 4 and 6). Significantly, the addition of an extra amphihaploid genome set from sexual *C. auratus* makes *C. gibelio* become amphitetraploid, which facilitates the proper synapsis and pairing of homologous chromosomes (fig. 4), and thereby enables equal homolog segregation and sexual reproductive recovery. Through interploidy hybridization, the produced novel amphitriploids return to triploidization and successively form two types of oocyte. Similarly, in naturally and experimentally produced F1 hybrids of unisexual *Cobitis*, two types of pachytene oocyte were observed, in which the vast majority (>95%) of oocytes containing a mixture of bivalents and univalents failed to develop further, whereas only the remaining oocytes formed double bivalents via premeiotic endoreplication and finally completed meiosis (Dedukh et al. 2021). Based on these observations, Dedukh et al. (2021) proposed that a pachytene checkpoint that involves DSB repair machinery (Roeder and Bailis 2000; Subramanian and Hochwagen 2014) might prevent developmental progression of the vast majority of oocytes and lead to their death. In the novel amphitriploids observed in the present study, type I oocytes gave priority to meiosis from sexual *C. auratus*, in which ∼50 SC bivalents were assembled, but the DSBs on the other unsynapsed 50 chromosomes were not completely repaired (fig. 6). Previous studies have shown that the incomplete repair of meiotic DSBs induces apoptosis (Pandey and Raghavan 2017; Dai et al. 2020), and we also detected massive apoptotic signals within type I oocytes (fig. 5). In contrast, type II oocytes adopt cytological mechanisms identical to unisexual *C. gibelio* to bypass meiosis and develop into mature eggs. Importantly, unisexual gynogenesis is stabilized in the gynogenetic amphitriploids produced by type II oocytes, as only type II oocytes are formed in the ovaries of gynogenetic amphitriploids (fig. 7). This led us question why type I oocytes disappear from the successive clonal offspring. As revealed in figures 4–7, type I oocytes entered apoptosis and failed to transmit genetic information to their offspring. Currently, it is not known why type II oocytes are able to bypass meiosis and develop into mature eggs, but one possible scenario may involve epigenetic regulation, which has been assumed in some unisexual species with high genetic similarity but show phenotypic variation (Albertini et al. 2019; Laskowski et al. 2019). Previous studies involving triploid unisexual dandelions (*T. officinale*) (Koen et al. 2010) and Iberian cyprinids (*S. alburnoides*) (Inácio et al. 2012) have suggested that hybridization and polyploidization may trigger immediate epigenetic changes. Considering that two types of oocyte seen in novel amphitriploids and the oocytes of gynogenetic amphitriploids should possess same genomic compositions, we hypothesize that epigenetic variations may underpin these differences in oocyte development fate and that the oocytes in gynogenetic amphitriploids may inherit the epigenetic variations from type II oocytes. A challenging future study would be to compare the differences in epigenetic states between the two types of oocyte. Single-cell epigenomics (Kelsey et al. 2017) will be able to open up this possibility.

The results of the current study give an interesting insight into sex-asymmetrical reproduction consequences in novel amphitriploids: the females of novel amphitriploids regain gynogenesis ability. However, most of the males showed arrested testes and the remaining produced abnormal sperm. These sex-specific differences have previously been observed in some unisexual complexes (Stöck et al. 2021), such as the *C. gibelio* complex (Wei et al. 2003) and the *Cobitis* complex (Park et al. 2011; Dedukh et al. 2020). In asexual triploid hybrids of Dojo loach (*Misgurnus anguillicaudatus*) (Kuroda et al. 2019), females generally reproduce clonally, and natural hybrid males are sterile, whereas sex-reversal males produce fertile sperm via premeiotic endoreplication like the females, implying that the capability of clonal gametogenesis might depend upon genetic sex determination. In contrast, in the *Cobitis* complex, when spermatogonia of sterile triploid males of *C. elongatoides-taenia-taenia* were transplanted into *C. elongatoides* females, the transplanted cells developed into clonal oocytes (Tichopád et al. 2022). This evidence indicates that significant similarities and differences regarding sex-asymmetrical consequences exist among different unisexual complexes, which awaits further investigation.

In conclusion, this study uncovers an efficient strategy for generating diverse clonal lineages in polyploid *Carassius* complex, which may enable gynogenetic *Carassius* avoid genomic decay and increase the potential evolvability. The current study broadens our understanding of the origin and maintenance of genetic diversity among unisexual species.

## Materials and Methods

### Maintenance of Fish

All experimental fish, including clone A^+^ and wild clone H of amphitriploid *C. gibelio* (*Cg*) (Wang et al. 2011; Gao et al. 2017), amphidiploid transparent *C. auratus* color variety (*Ca*), synthetic amphitetraploid male (SA4n♂), and novel amphitriploid (NA3n) (Lu et al. 2021) were raised and collected from the National Aquatic Biological Resource Center, NABRC. Before being sampled, the fish were anesthetized with styrylpyridine (30 mg–50 mg/l; Aladdin, China). All procedures were performed with the approval of the Animal Care and Use Committee of the Institute of Hydrobiology, Chinese Academy of Sciences.

### Chromosome Preparation and Fluorescence In Situ Hybridization (FISH)

The preparation of metaphase chromosomes was performed as described (Zhu and Gui 2007; Li et al. 2016b). Six DNA probes were used to perform FISH analysis according to standard procedures as described previously. The BAC-DNAs marking chromosome 5A (Chr5A), Chr5B, Chr17A, and Chr17B (Li et al. 2014; Mou et al. 2021) were labeled by DIG-Nick Translation Mix and Biotin-Nick Translation Mix (Roche), respectively. To investigate the sex determination system of the novel amphitriploids and its offspring, the fragments specific to sex chromosomes (Chr22B) were amplified by PCR and labeled by DIG-Nick Translation Mix (Roche, Switzerland) (Lu et al. 2021), and the genomic DNA of *C. auratus* labeled by Biotin-Nick Labeling System were used to distinguish X and Y chromosomes. The chromosomes were stained with DAPI (Sigma, USA). The sections were observed and photographed by Zeiss Axio Imager2 (Analytical & Testing Center, IHB, CAS).

### TF Electrophoresis and Microsatellite Analysis

According to the rivanol treatment procedure, TF was isolated from serum and separated by 10% polyacrylamide gel electrophoresis (Yang and Gui 2004; Li and Gui 2008). Microsatellite analysis was conducted according to the standard procedures as described previously (Zhao et al. 2021), 15 pairs of microsatellite primers were used to exhibit genetic diversity in the novel amphitriploids.

### Analysis of Chromosome Composition in Novel Amphitriploids by Whole-genome Resequencing

#### Whole-Genome Resequencing

The caudal fins of ten novel amphitriploid individuals (NA3n), as well as the maternal *C. auratu*s ♀ (*Ca*♀), and the parents *C. gibelio* ♀ and *C. auratu*s ♂ (*Cg*♀ and *Ca*♂) of the paternal fertile synthetic amphitetraploid male were sampled for genomic DNA extraction using a Genomic DNA Extraction Kit (Promega, USA) following the manufacturer’s instructions. The resequencing was performed on the DNBSEQ-T7 (MGI, China) platform and the NovaSeq 6000 platform (Illumina, USA) (GenBank accession: PRJNA814448).

#### SNP Calling

The raw reads were filtered using Fastp-0.23.0 (supplementary table S1, Supplementary Material online), with a required length of 50, and a default qualified quality phred cutoff of 15, and a default unqualified percent limit of 40. Clean reads were aligned to the reference genome of *C. auratus* (BioProject ID: PRJNA546444) (Wang, Li, Xu et al. 2022) using the online software BWA-0.7.17. After preprocessing the bam files by marking duplicates, we called the SNP and realigned indels using GATK4 (Lu et al. 2019). To analyze the genome composition of the novel amphitriploids, only biallelic SNPs were retained, and these were filtered using the function SelectVariants in GATK4 with the following parameters: filter-expression “QD < 2.0 || FS > 60.0 || MQ < 40.0 || MQRankSum < −12.5 || ReadPosRankSum < −8.0.”

#### Genotyping of *C. gibelio* and *C. auratus* Chromosomes in the Novel Amphitriploids

Genotyping was conducted as described in *Caenorhabditis* nematodes (Lamelza et al. 2019). Considering the ploidy differences between the samples, effective loci for genotyping must meet the following criteria:

1. Genotype of *Cg*♀, *Ca*♀, and *Ca*♂ are all homozygous.
2. Genotype of *Ca*♀ and *Ca*♂ are the same.
3. Genotype of *Cg*♀ and *Ca* (*Ca*♀ and *Ca*♂) are different.
4. Genotype of NA3n is heterozygous.

A total of 589,200–625,938 loci (supplementary fig. S3, Supplementary Material online) that satisfied all of the above criteria were used to determine the chromosome composition in ten individuals of the novel amphitriploids.

#### SNP Frequency Calculations

The chromosomal genotypes of the novel amphitriploids were identified according to the mean SNP frequency of the *C. gibelio* genotype and the *C. auratus* genotype. Mean SNP frequencies were calculated in 1 Mb windows across the whole genome or 100 kb windows across one chromosome. A scatter diagram was draw according to the mean SNP frequency of the *C. gibelio* genotype and the *C. auratus* genotype. Similar to that reported in *Caenorhabditis* nematodes (Lamelza et al. 2019), the distribution of SNP frequencies in the novel amphitriploids was skewed toward the *C. auratus* genotype, because the reads derived from *C. auratus* could be more easily mapped to the reference genome of *C. auratus*.

### Fertility Assessment of Novel Amphitriploid Females

Five groups of novel amphitriploids were synthetized by mating a fertile amphitetraploid male with five *C. auratus* females. The sex ratio was calculated via secondary sex characteristics and gonad observation. Histological analysis of gonads was performed as previously described (Takemoto et al. 2020). To test the fertility of the novel amphitriploid females, two different crossed combinations (supplementary table S6, Supplementary Material online) were performed between novel amphitriploid females and *C. auratus* or *C. carpio* males (NA3n♀ × *Ca*♂ and NA3n♀ × *Cc*♂). The crossed combination between female and male *C. auratus* (*Ca*♀ × *Ca*♂) was performed as a control. Artificial fertilization, larval hatching, and the calculation of fertilization rate and hatching rate were performed as previously described (Lu et al. 2021).

### Observation of Nuclear Events During Meiosis I and Fertilization Process

Females were intraperitoneally injected with a mixture of acetone-dried carp pituitary, human chorionic gonadotropin (HCG), and LRH-A to induce ovulation. According to the oocyte developmental stages in relation to meiosis in teleost fish (Lubzens et al. 2010), the metaphase, and anaphase of meiosis I were staged. At 6 h (23°C) postinjection (hpi), the GV broke down and the primary oocytes entered into the metaphase I at 7 hpi. Subsequently, the first polar body was excluded (7.5 hpi) and the matured oocytes (eggs) were arrested at metaphase II (8 hpi). The primary oocytes at 7–7.5 hpi and the fertilized eggs were stained by DAPI and the dynamics of the nuclear events during metaphase I, anaphase I, and fertilization were observed as described (Zhu et al. 2018). In brief, the primary oocytes of *C. gibelio*, *C. auratus*, and the novel amphitriploid (NA3n), as well as the fertilized eggs of the crossed combination (including *Cg*♀ × SA4n♂, NA3n♀ × *Ca*♂ and NA3n♀ × *Cc*♂), were digested by 0.25% trypsin (VWR, USA) to remove their shells. The oocytes and fertilized eggs at different developmental stages were fixed with 4% paraformaldehyde in phosphate buffer saline (PBS) at 4°C overnight. After washing with PBS three times, the nuclei were stained by DAPI and the images were acquired under Leica SP8 DLS confocal microscopy (Analytical & Testing Center, IHB, CAS).

### Measurement of DNA Content via Flow Cytometry

DNA content of blood was measured by Cytoflex S Flow Systems (Beckman, USA) as described previously (Lu et al. 2021) and the ploidy levels were determined according to the control.

### Preparation of Meiotic Oocyte Chromosomal Spreads and Immunostaining

Three females of the novel amphitriploid or gynogenetic amphitriploid at different developmental stages (30, 50, 70, 90, 110, 130, and 150 dph) were randomly sampled for oocyte chromosomal spreads and apoptosis detection. Meiotic oocyte chromosomal spreads were prepared from ovaries by an improved procedure and immunostaining was performed as described previously (Araya-Jaime et al. 2015; Takemoto et al. 2020). In brief, half of the ovaries were minced in 0.75% saline solution with a 3-fold volume of ovary tissue. After natural precipitation for 5 min, the cell suspension was transferred into a new tube and then a 3-fold volume of hypotonic solution (KCL, 0.075 mol/l) was added. Two types of chromosomal spreads were prepared. Half of the cell suspension was immediately placed onto clean slides and dried at room temperature, in which the nuclei were fully spread on the slide and used to clearly count the numbers of SC. This nucleus spreading can be used directly for immunostaining without antigenic repair. The other half of the cell suspension was maintained hypotonically for 30 min, then a 1/3 volume of 4% paraformaldehyde solution was added to fix the nuclei. Then, the cell suspension was placed onto clean slides and dried at room temperature, in which the nuclei were slightly spread on the slide and used to show subcellular localization of the proteins in the nucleus with the original morphology of the SC and nuclear polarity. The slides were used for immunostaining as described previously (Araya-Jaime et al. 2015). All antibodies and dilutions used are listed in supplementary table S9, Supplementary Material online. The images were acquired under Leica STELLARIS 8 FALCON confocal microscopy (Analytical & Testing Center, IHB, CAS).

### Apoptosis Detection

Half of the ovaries were fixed in 4% paraformaldehyde at 4°C overnight. The fixed samples were dehydrated in an ethanol series (70%, 80%, 90%, and 100%), with methyl benzoate and Lemosol (Wako) and then embedded in paraffin. TUNEL staining and HE staining were performed, respectively, as described previously (Mei et al. 2008; Zhang et al. 2021) with 9-μm-thick sections.

### Isolation of GV and Bivalent Spread

Four hours (23°C) after intraperitoneal injection with a mixture of acetone-dried carp pituitary, HCG, and LRH-A, the eggs were collected to isolate GVs using fine forceps in according to a previous report (Gall and Wu 2010). GVs were transferred with a pipette to glass-bottom dishes containing hypotonic solution (KCL, 0.075 mol/l), then transferred to clean slides, which were dried at room temperature. The bivalents were fully spread on the slides. The chromosomes were stained with DAPI and photographed under Leica SP8 DLS confocal microscopy (Analytical and Testing Center, IHB, CAS).

### Identification of Parental Chromosomes and Genetic Similarity Analysis in Three Successive Amphitriploid Generations

#### Whole-Genome Resequencing

The caudal fins of ten individuals of the gynogenetic amphitriploid (GA3n) and cloned amphitriploid (CA3n) were sampled to extract genomic DNA. The whole-genome resequencing was performed on the MGI (DNBSEQ-T7, China) platform (GenBank accession: PRJNA814448).

#### Genotyping of Parental Chromosomes in Three Amphitriploid Generations

Reads filtering (supplementary table S1, Supplementary Material online) and SNP calling were performed as described above. Effective loci that were homozygous and differed between the parents of each generation were used for genotyping in the amphitriploid offspring. The mean SNP frequencies of the paternal genotype and maternal genotype were calculated in 1 Mb windows across the reference genome of *C. auratus*, and the composition of parental chromosomes in ten individuals from three amphitriploid generations were estimated according to the mean SNP frequency.

#### Genetic Diversity Analysis

The genetic diversity among each amphitriploid generation was quantified using an IBS analysis. IBS analysis was performed according to the dataset of SNPs using PlinK as previously described (Barley et al. 2022). Heat maps were drawn using the R package “pheatmap.”

## Supplementary Material

msac188_Supplementary_Data

## Data Availability

The whole-genome assemblies of *C. auratus* are deposited at GenBank database under the accession number of PRJNA546444 (BioSample SAMN11978330). The raw reads of 30 amphitriploid individuals from NA3n, GA3n, CA3n and their parents have been submitted to the NCBI BioProject database under accession number PRJNA814448. And the other data are available within the paper and its supplementary materials.
