# Supplementary material for: Changes in Ploidy Drive Reproduction Transition and Genomic Diversity in a Polyploid Fish Complex
Source: Mol Biol Evol. 2022 Sep 3;39(9):msac188. doi: 10.1093/molbev/msac188 (PMC9486886; doi:10.1093/molbev/msac188)
Supplement: msac188_Supplementary_Data [file msac188_supplementary_data.pdf]

Supplementary information

Supplementary Figure 1. FISH analysis of *Cgviperin-A*-BAC-DNA and *Cgviperin-B*-BAC-DNA on metaphase Chr A17 and Chr B17. BAC DNA was labeled with DIG (green) or biotin (red) respectively and all metaphase chromosomes (blue) were counterstained with DAPI.

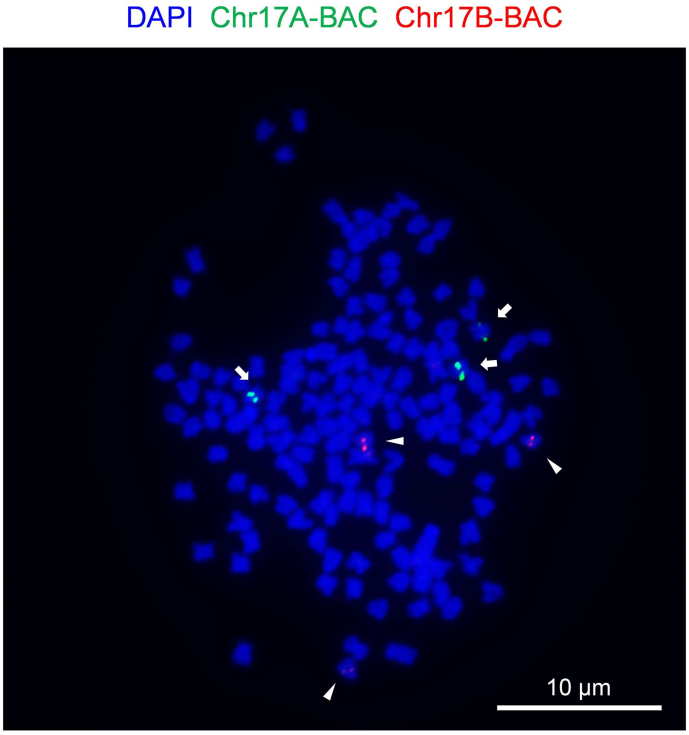

Supplementary Figure 2. Microsatellite genotypes of NA3n with their parents amplified by the primers of YJ0001, YJ0002, YJ0010, YJ0020, YJ0022, YJ0025, YJ0039, YJ0040, YJ0042, MFW1, MFW19, and MFW23. *Ca*, *C. auratus*; NA3n, novel amphitriploid; SA4n, synthetic amphitetraploid; ♀, female; ♂, male; I–XI, genotype 1 to 11.

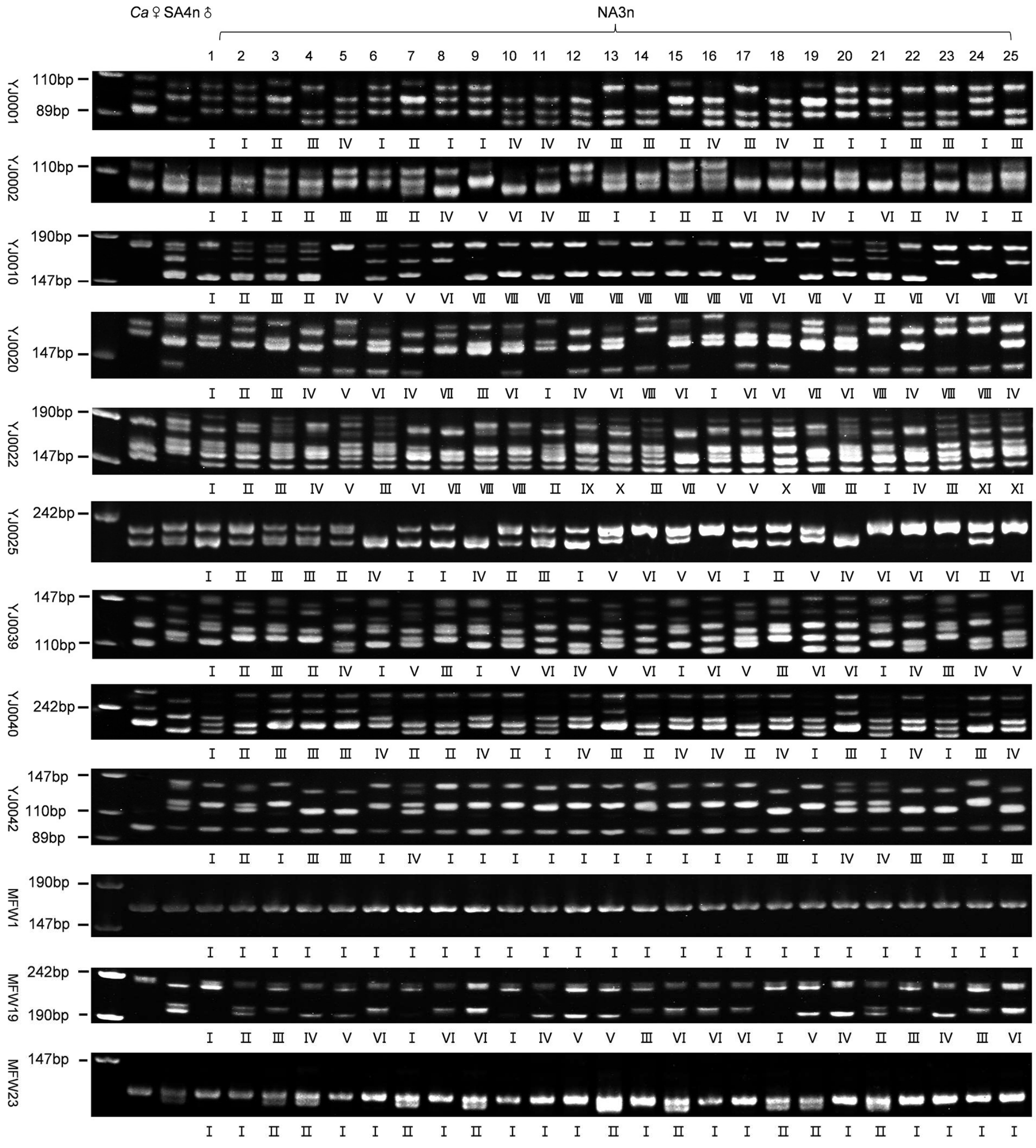

Supplementary Figure 3. Screening strategy used to identify effective loci (A) and schematic diagram how to perform chromosome genotyping according to SNP frequency of effective loci (B).

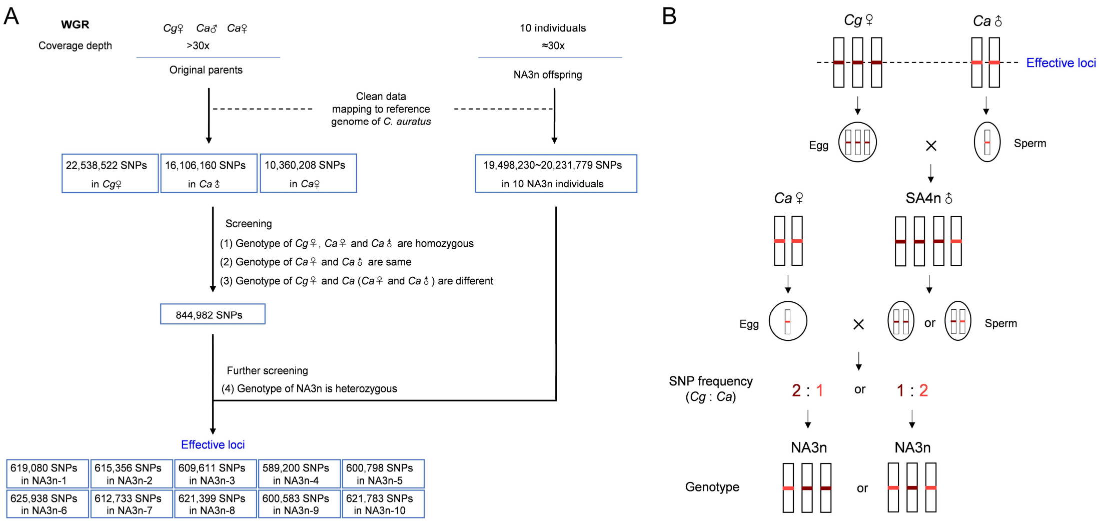

Supplementary Figure 4. Whole-genome genotyping data from 10 individuals of novel amphitriploid (NA3n). Each point represents the average SNP frequency in 1-Mb windows ordered across the assembled genome of *C. auratus* (Chr1A and Chr1B to Chr25A and Chr25B). Dark red, bright red, dark yellow, and bright yellow represent the genotypes of *C. gibelio* and *C. auratus* in subgenomes A and B, respectively. We found a few homologous chromosome groups to show unusual SNP frequency of *C. gibelio* and *C. auratus* genotypes in eight individuals of the novel amphitriploid, implying that these homologous chromosome groups have lost or obtained one homologous chromosome from *C. gibelio* or *C. auratus*. For example, NA3n-3 had three homologous chromosome groups with deviating expected SNP frequency. Both Chr10A and Chr13B had approximate SNP frequency of 0.5 (1/2), implying that NA3n-3 might only inherit one homologous chromosome of them from *C. gibelio* and *C. auratus*, respectively. In Chr18B, an approximate SNP frequency of 0.25 (1/4) for *C. gibelio* and 0.75 (3/4) for *C. auratus* were inferred that NA3n-3 might inherit three homologous chromosomes from *C. auratus* and one homologous chromosome from *C. gibelio*, respectively.

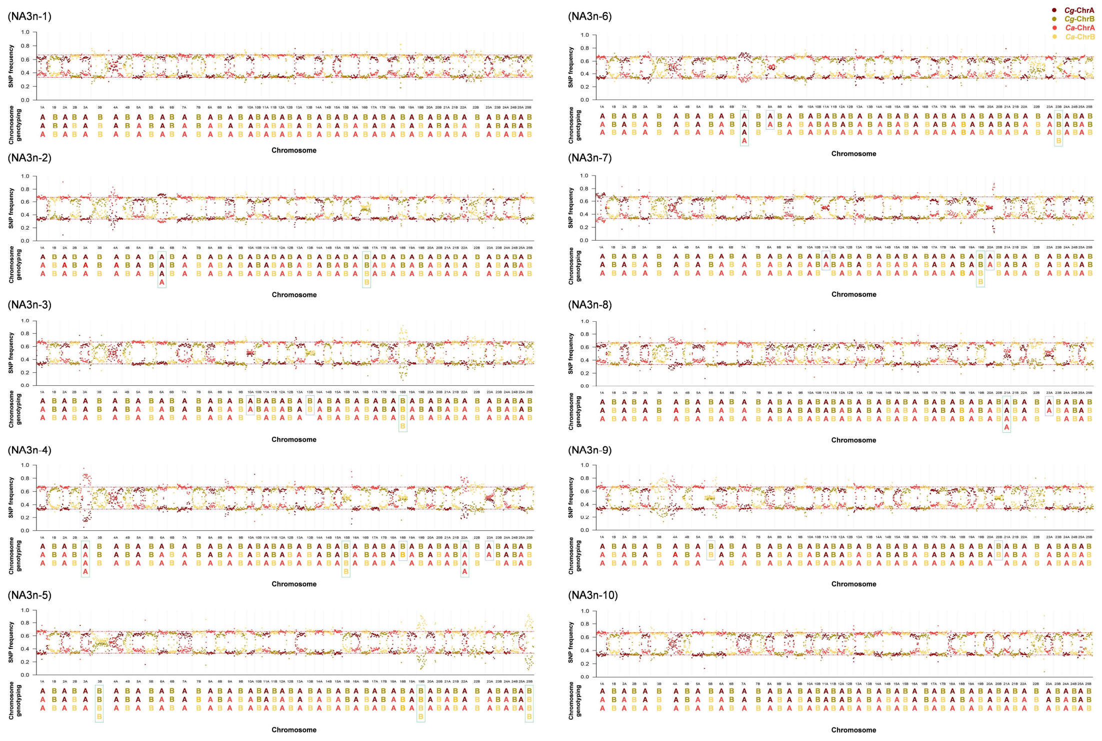

Supplementary Figure 5. Genotyping of all 50 chromosomes in NA3n-1. Each point represents the average SNP frequency in 100-kb windows ordered along the chromosome.

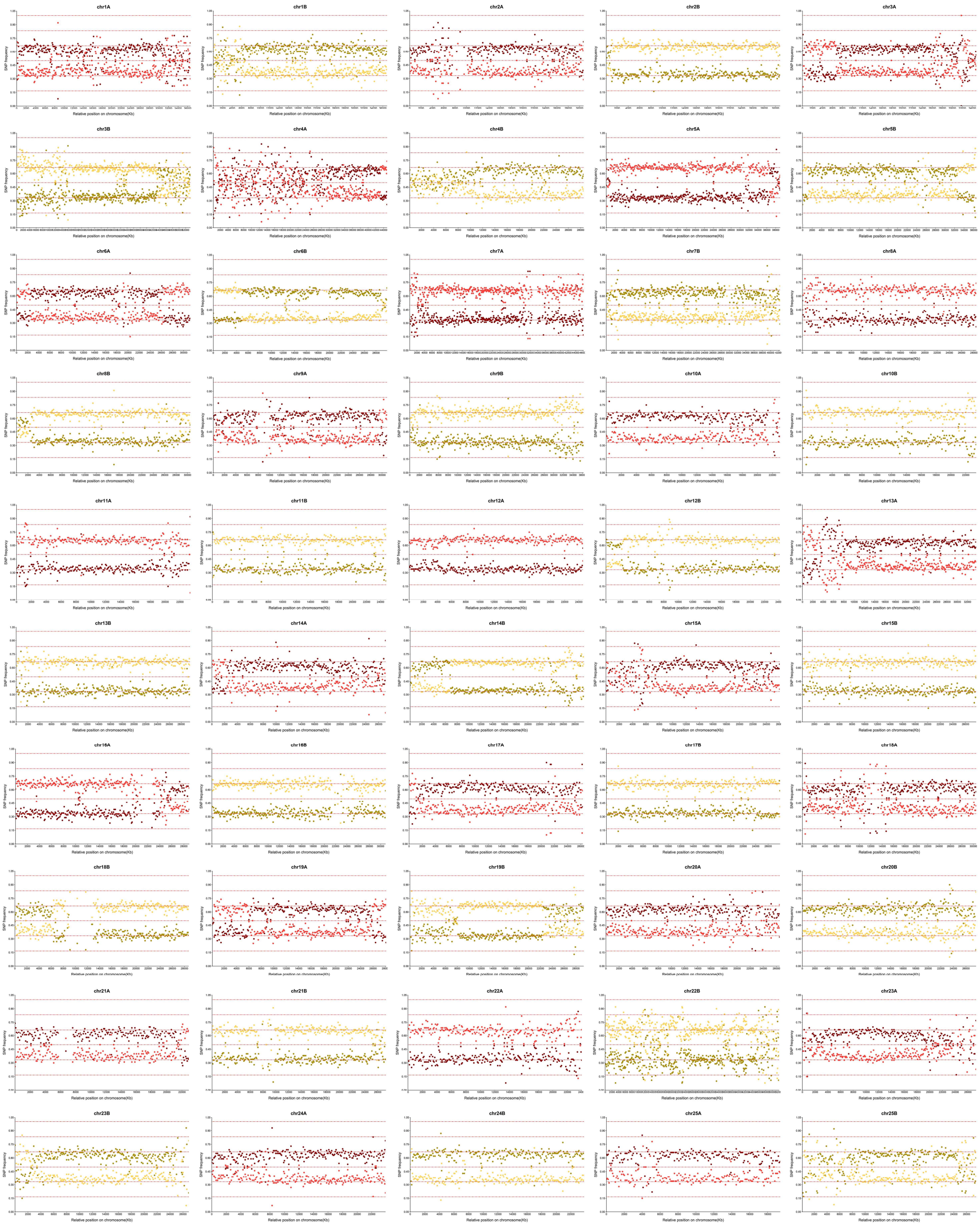

Supplementary Figure 6. Box-plot of *Cg*-derived SNP frequency statistics of all 50 chromosomes in 10 novel amphitriploids. The occurrence of homologous recombination would significantly increase the fluctuation range of *Cg*-derived SNP frequency, such as Chr6A of NA3n-1 and Chr6B of NA3n-2.

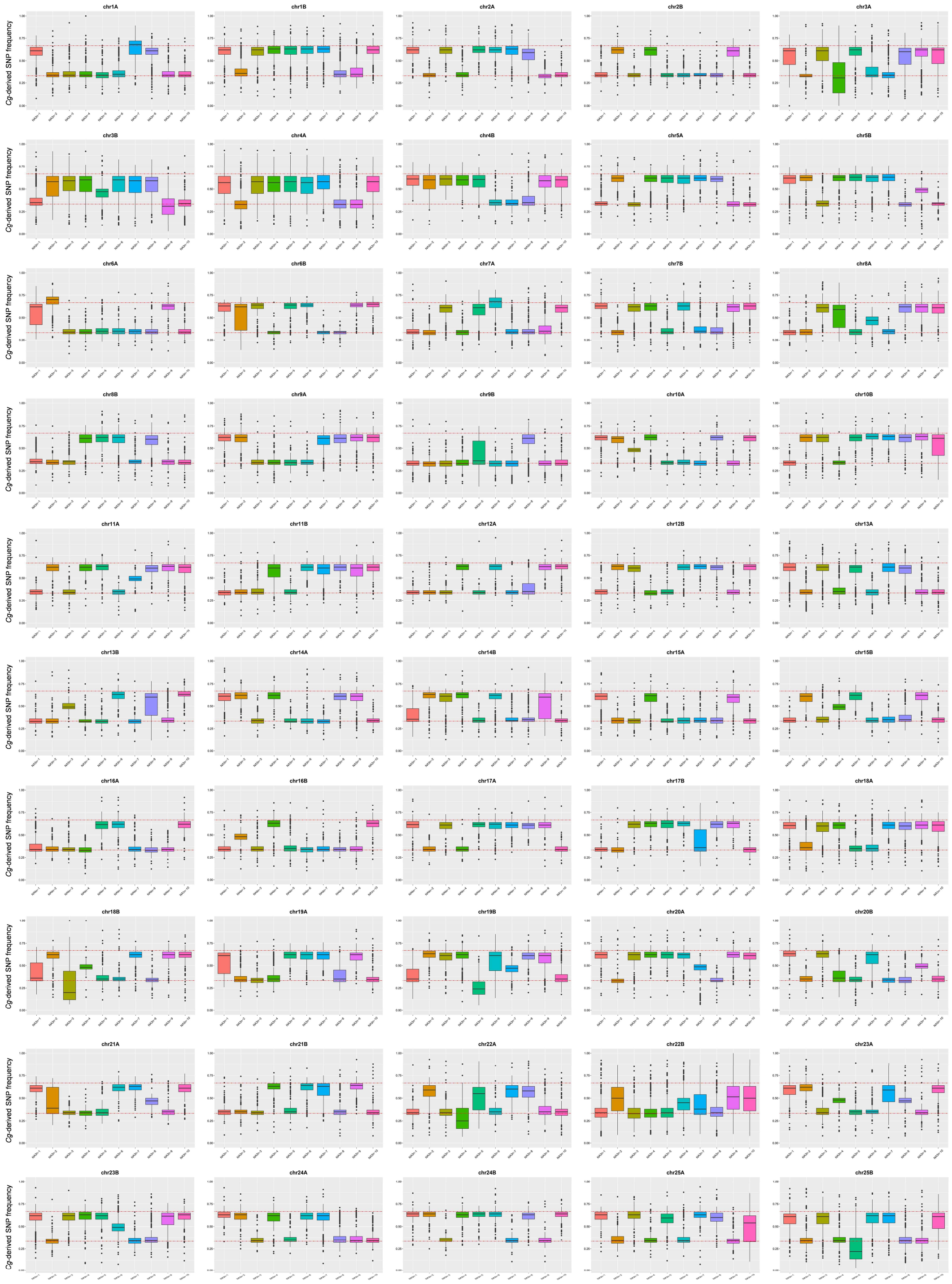

Supplementary Figure 7. Four unisexual amphitriploid clones were reproduced by the recovered gynogenesis.

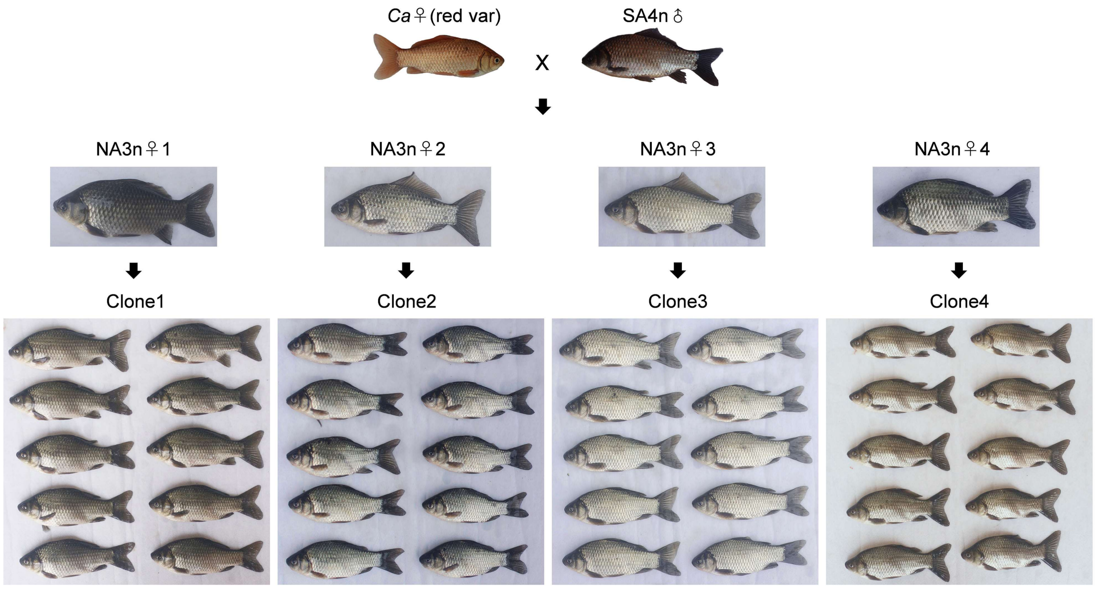

Supplementary Figure 8. A few of spermatocyte microspreads showing univalents (A) and tetravalent (B) in the synthetic amphitetraploid male (SA4n♂). Chromosomal microspreads of spermatocytes were coimmunostained by anti-Sycp1 (red) and anti-Sycp3 (green) antibodies. Asterisks indicate univalent SC.

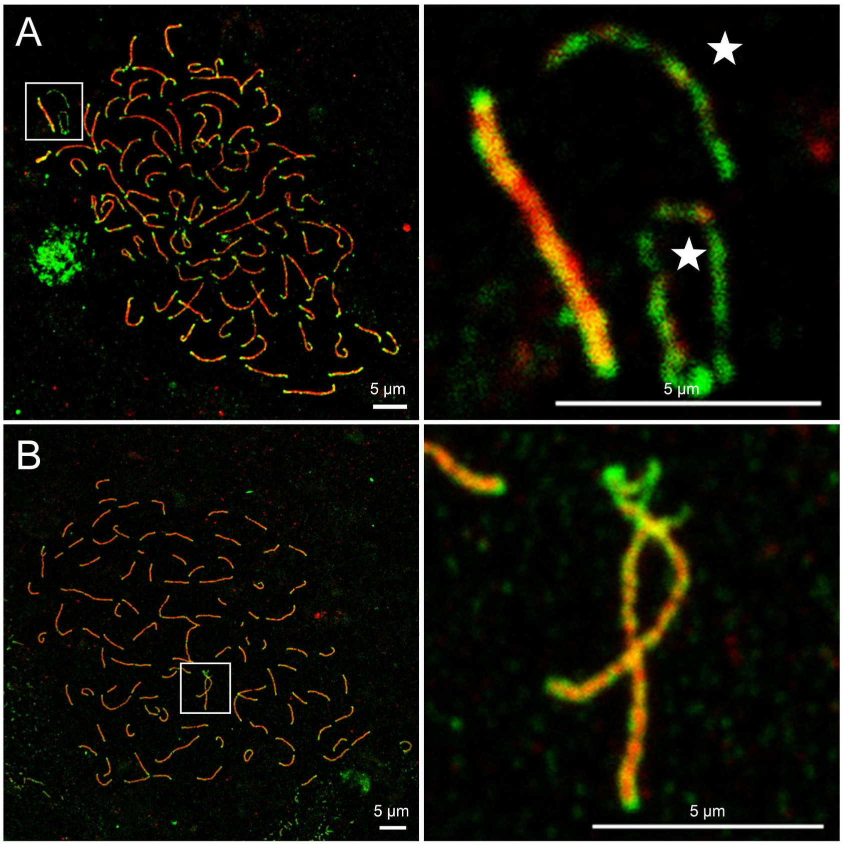

Supplementary Figure 9. Ameiotic process of *C. gibelio* wild clone H. A-B, DSB formation and repair marked by γH2AX (A) and Rad51 (B). Chromosomal spreads of primary oocytes were coimmunostained by anti-γH2AX (A) (red) or anti-Rad51 (B) (red) with anti-Sycp1 (magenta) and Sycp3 (green) antibodies. Nuclei were stained with DAPI (blue). Females of clone H (n = 3) at 90 dph were sampled for oocyte chromosomal spreads. C, Recombination sites were identified by anti-Mlh1 antibody (red) at the pachytene stage. SCs were visualized by anti-Sycp1 (magenta) and anti-Sycp3 (green) antibodies. Nuclei were stained with DAPI (blue). D, DAPI-stained chromosome spread of germinal vesicle at diakinesis. 50 eggs from clone H (n = 3) were collected to isolate GVs. E, Nuclear behaviors at the metaphase I and anaphase I stage. 50 eggs from clone H (n = 3) were collected for cytological observation. F, Nuclear behaviors in the fertilized eggs stained by DAPI. The fertilized eggs of clone H♀ mating with *C. auratus*♂ and *C. gibelio*♂ of GSD. Thin arrows, thick arrows, and arrowheads indicate sperm nucleus, female pronucleus, and second polar-body, respectively. Asterisks indicate the nucleus of zygote after the first mitosis. The corresponding time after fertilization is showed above.

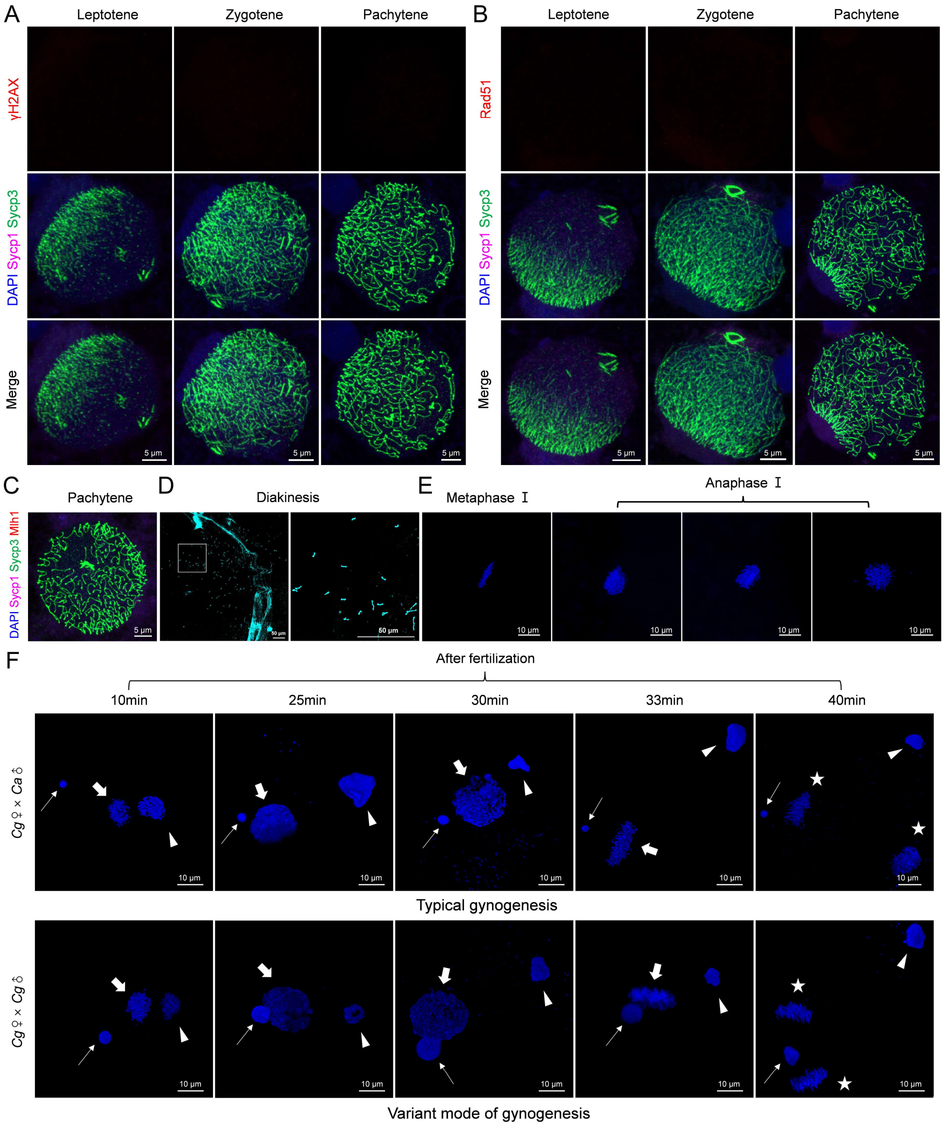

Supplementary Figure 10. Sporadic Rad51 signals were detected from a small number of oocytes of clone A$^+$ (10%, n = 50) and clone H (14%, n = 50) of *C. gibelio* as well as novel amphitriploid type Ⅱ oocytes (22%, n = 50). *Cg*, *C. gibelio*; NA3n, novel amphitriploid; ♀, female.

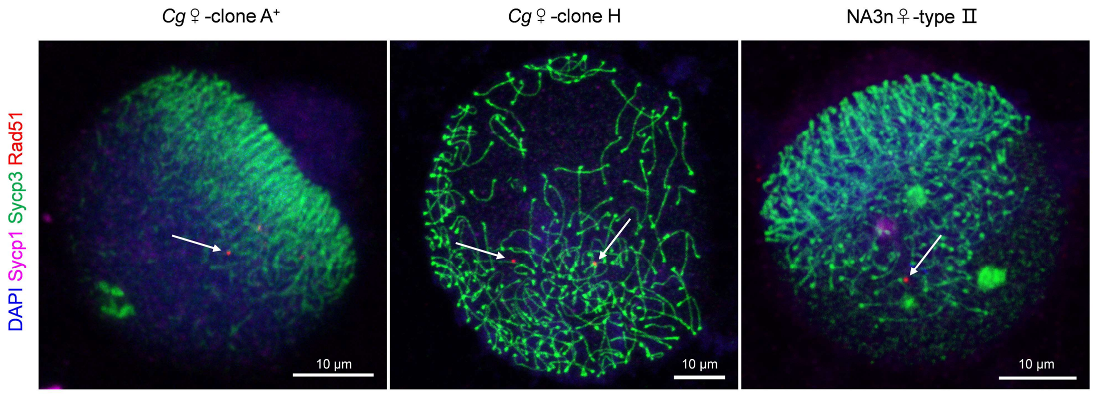

Supplementary Figure 11. A special microspread showing one Mlh1 signal in a type Ⅱ oocyte of novel amphitriploid. Recombination site and SCs were visualized by anti-Mlh1 antibody (red), anti-Sycp1 (magenta) and anti-Sycp3 (green) antibodies. Nuclei were stained with DAPI (blue).

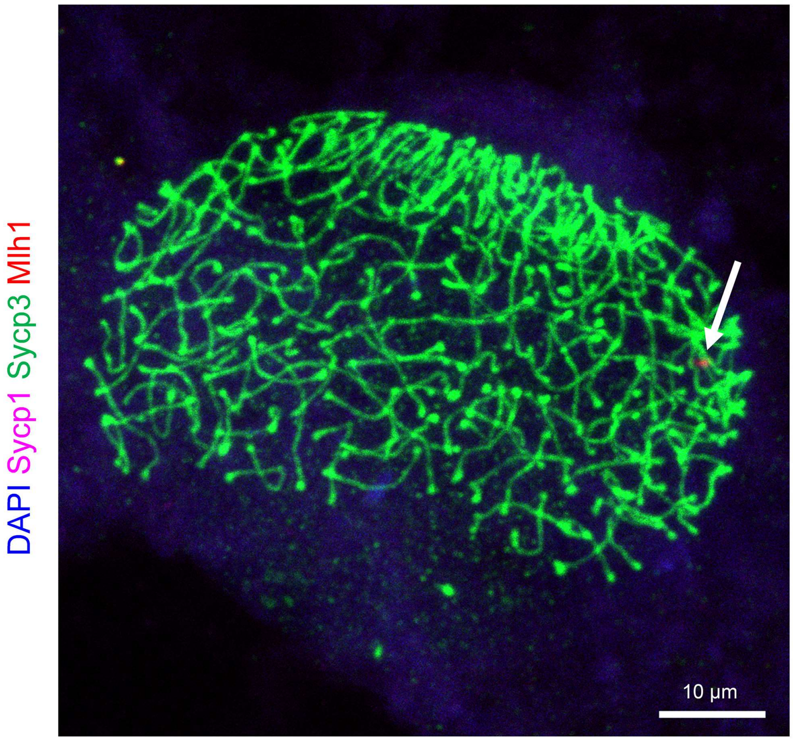

Supplementary Figure 12. Compositional analysis of parental chromosome sets in another nine individuals of novel amphitriploid (NA3n) by genome resequencing. Each point represents the average SNP frequency in 1-Mb windows ordered across the assembled genome of *C. auratus*. Blue and red represent the chromosome genotypes of maternal and paternal parent. A few homologous chromosomes group had unusual SNP frequency of maternal and paternal genotype, such as 0.5 (1/2) (i.e. Chr10A of NA3n-3 and Chr18B of NA3n-4), 0.25 (1/4) and 0.75 (3/4) (i.e. Chr15B of NA3n-4 and Chr3B of NA3n-5), implying that these chromosomes have lost or obtained one homolog from maternal or paternal parents.

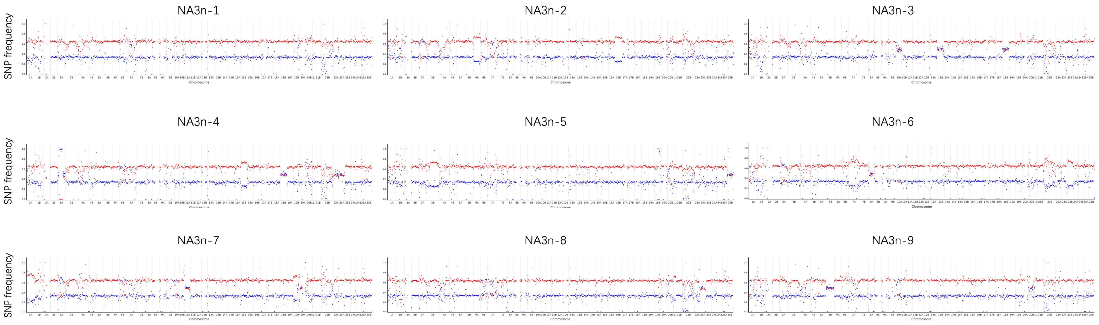

Supplementary Figure 13. Compositional analysis of parental chromosome sets in another nine individuals of gynogenetic amphitriploids (GA3n) by genome resequencing. Each point represents the average SNP frequency in 1-Mb windows ordered across the assembled genome of *C. auratus*. Blue and red represent the chromosome genotypes of maternal and paternal parent.

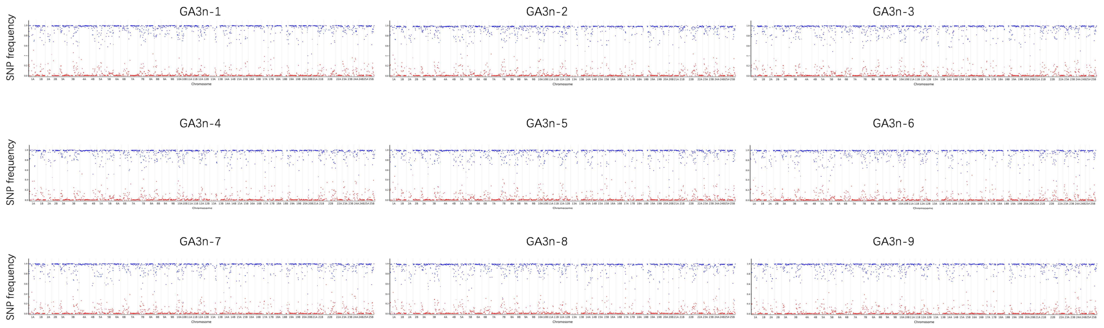

Supplementary Figure 14. Compositional analysis of parental chromosome sets in another nine individuals of the cloned amphitriploids (CA3n) by genome resequencing. Each point represents the average SNP frequency in 1-Mb windows ordered across the assembled genome of *C. auratus*. Blue and red represent the chromosome genotypes of maternal and paternal parent.

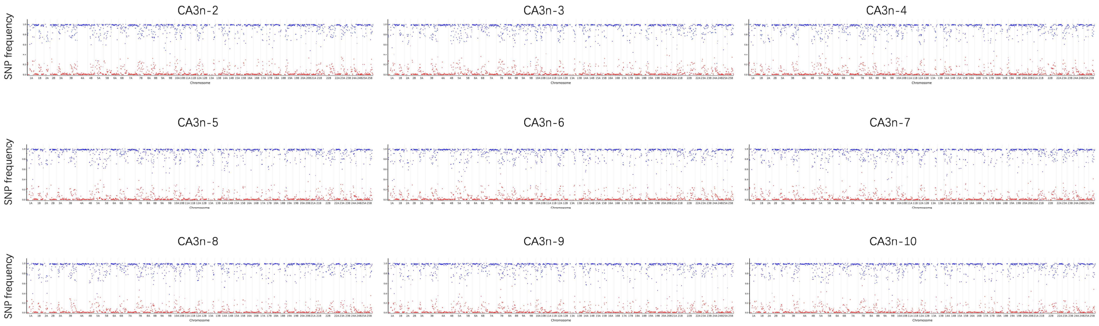

Supplementary Table 1. Summary statistics of resequencing data.

Platform	Sample	Raw_Base	Clean_Base	Clean_Base_Percent	GC_Content	>Q20	>Q30
Illumina (NovaSeq 6000)	*Cg*♀ (Maternal parent of SA4n♂)	260666991300(260.67G)	254567689490(254.57G)	97.66%	39.30%	97.96%	94.36%
	Ca♂ (Paternal parent of SA4n♂)	174174669900(174.17G)	158043765434(158.04G)	90.74%	38.11%	95.63%	89.53%
	SA4n♂ (Paternal parent of NA3n)	110066770500(110.07G)	108397284849(108.40G)	98.48%	39.61%	97.85%	93.62%
MGI (DNBSEQ-T7)	*Ca*♀ (Maternal parent of NA3n)	71086595400(71.09G)	70367715495(70.37G)	98.99%	37.79%	97.09%	91.55%
	NA3n-1	77725109381(77.73G)	77139120661(77.14G)	99.25%	37.72%	96.70%	90.53%
	NA3n-2	68695314855(68.70G)	68040279423(68.04G)	99.05%	37.91%	96.42%	89.76%
	NA3n-3	67425381891(67.43G)	66926439428(66.93G)	99.26%	37.72%	96.74%	90.71%
	NA3n-4	65434768065(65.43G)	64946430585(64.95G)	99.25%	37.75%	96.71%	90.53%
	NA3n-5	68056438257(68.06G)	67507322393(67.51G)	99.19%	37.71%	96.55%	90.17%
	NA3n-6	70204229509(70.20G)	69644028536(69.64G)	99.20%	37.78%	96.58%	90.21%
	NA3n-7	67287735805(67.29G)	66825496575(66.83G)	99.31%	37.90%	96.74%	90.68%
	NA3n-8	70616041943(70.62G)	70110269481(70.11G)	99.28%	37.91%	95.19%	86.64%
	NA3n-9	72705334472(72.71G)	72159335987(72.16G)	99.25%	37.84%	96.18%	89.08%
	NA3n-10 (Maternal parent of GA3n)	67711962600(67.71G)	66988592643(66.99G)	98.93%	37.71%	96.82%	90.71%
	Ca♂ (Paternal parent of GA3n and CA3n)	112818278400(112.82G)	111627693285(111.63G)	98.94%	37.75%	96.78%	90.64%
	GA3n-1	72628535161(72.63G)	72130933319(72.13G)	99.31%	37.63%	96.29%	89.29%
	GA3n-2	81208861643(81.21G)	80699160697(80.70G)	99.37%	37.79%	96.19%	89.15%
	GA3n-3	75905361574(75.91G)	75367170664(75.37G)	99.29%	37.72%	96.20%	89.08%
	GA3n-4	70212383326(70.21G)	69112470349(69.11G)	98.43%	37.93%	95.70%	88.28%
	GA3n-5	57302097615(57.30G)	56910392382(56.91G)	99.32%	37.63%	95.78%	88.10%
	GA3n-6	62875385998(62.88G)	62468382419(62.47G)	99.35%	37.71%	96.09%	88.92%
	GA3n-7	57411432637(57.41G)	56980393677(56.98G)	99.25%	37.61%	96.10%	89.00%
	GA3n-8	67166250020(67.17G)	66691241096(66.69G)	99.29%	37.72%	96.10%	88.96%
	GA3n-9	58542963335(58.54G)	58133384854(58.13G)	99.30%	37.56%	95.92%	88.49%
	GA3n-10 (Maternal parent of CA3n)	55134514200(55.13G)	54570000410(54.57G)	98.98%	37.71%	96.56%	90.10%
	CA3n-1	60593711139(60.59G)	60234892575(60.23G)	99.41%	37.65%	95.95%	88.56%
	CA3n-2	64476717439(64.48G)	64045448233(64.05G)	99.33%	37.56%	95.85%	88.36%
	CA3n-3	91467080527(91.47G)	90807608309(90.81G)	99.28%	37.65%	95.95%	88.49%
	CA3n-4	92169370312(92.17G)	91496269706(91.50G)	99.27%	37.66%	95.90%	88.40%
	CA3n-5	73433990319(73.43G)	72917364783(72.92G)	99.30%	37.64%	96.04%	88.79%
	CA3n-6	75526323437(75.53G)	74971290420(74.97G)	99.27%	37.66%	96.02%	88.74%
	CA3n-7	68992933318(68.99G)	68516393015(68.52G)	99.31%	37.45%	95.72%	88.10%
	CA3n-8	71412300969(71.41G)	70957302606(70.96G)	99.36%	37.55%	95.83%	88.39%
	CA3n-9	73150742865(73.15G)	72611791064(72.61G)	99.26%	37.63%	96.20%	89.16%
	CA3n-10	74527986389(74.53G)	73991399848(73.99G)	99.28%	37.72%	96.41%	89.78%

Supplementary Table 2. Average ratio of non-recombination and recombination homolog groups in 10 individuals of the novel amphitriploid (NA3n).

Sample	Ratio of non-recombination	Ratio of recombination
NA3n-1	48.0%	52.0%
NA3n-2	57.4%	42.6%
NA3n-3	45.7%	54.3%
NA3n-4	52.3%	47.7%
NA3n-5	54.3%	45.7%
NA3n-6	56.5%	43.5%
NA3n-7	54.3%	45.7%
NA3n-8	40.4%	59.6%
NA3n-9	45.7%	54.3%
NA3n-10	51.0%	49.0%
Average ratio	50.6%	49.4%
Standard deviation	5.5%	5.5%

Supplementary Table 3. Average ratio of *Cg*-derived and *Ca*-derived chromosomes in 10 individuals of the novel amphitriploid (NA3n).

Sample	Ratio of *Cg*-derived chromosome	Ratio of *Ca*-derived chromosome
NA3n-1	51.3%	48.7%
NA3n-2	48.0%	52.0%
NA3n-3	49.0%	51.0%
NA3n-4	51.0%	49.0%
NA3n-5	48.4%	51.6%
NA3n-6	53.0%	47.0%
NA3n-7	49.7%	50.3%
NA3n-8	50.0%	50.0%
NA3n-9	51.7%	48.3%
NA3n-10	52.0%	48.0%
Average ratio	50.4%	49.6%
Standard deviation	1.7%	1.7%

Supplementary Table 4. Sex ratio of the novel amphitriploid (NA3n) offspring from five groups.

Crossed combination	Group	♀	♂	Sex ratio (♀/♂)
Ca♀ × SA4n♂	*Ca*♀1 × SA4n♂1	43	38	1.13
	Ca♀2 × SA4n♂1	46	49	0.94
	Ca♀3 × SA4n♂1	44	42	1.05
	Ca♀4 × SA4n♂1	40	42	0.95
	Ca♀5 × SA4n♂1	45	45	1.00

Supplementary Table 5. Ratio of normal ovary in five groups of the novel amphitriploid.

Group	Ovary		
	Normal ovary	Arrested ovary	Ratio of normal ovary
1	36	7	83.7%
2	35	11	76.1%
3	33	11	75.0%
4	32	8	80.0%
5	39	6	86.7%
	Average ratio		80.3%
	Standard deviation		5.0%

Supplementary Table 6. Fertilization rate and survival rate of three crossed combinations.

Crossed combination		Fertilization rate	Survival rate
1	NA3n♀1 × *Ca*♂1	86.9%	92.7%
	NA3n♀2 × *Ca*♂1	87.6%	96.1%
	NA3n♀3 × *Ca*♂1	81.9%	92.1%
	NA3n♀4 × *Ca*♂1	91.2%	90.1%
	NA3n♀5 × *Ca*♂1	85.2%	92.5%
	Average rate	86.6%	92.7%
	Standard deviation	3.4%	2.2%
2	NA3n♀1 × *Cc*♂1	89.1%	94.4%
	NA3n♀2 × *Cc*♂1	82.6%	94.9%
	NA3n♀3 × *Cc*♂1	80.5%	95.2%
	NA3n♀4 × *Cc*♂1	84.1%	90.6%
	NA3n♀5 × *Cc*♂1	85.6%	87.4%
	Average rate	84.4%	92.5%
	Standard deviation	3.2%	3.4%
3（control)	*Ca*♀1 × *Ca*♂1	93.1%	96.2%
	Ca♀2 × *Ca*♂1	89.5%	97.3%
	Ca♀3 × *Ca*♂1	88.4%	95.0%
	Ca♀4 × *Ca*♂1	87.9%	94.2%
	Ca♀5 × *Ca*♂1	92.5%	92.7%
	Average rate	90.3%	95.1%
	Standard deviation	2.4%	1.8%

Supplementary Table 7. Fluorescent values of blood cells from 10 offspring of NA3n♀1 × *Ca*♂1 and NA3n♀1 × *Cc*♂1.

Crossed combination	Sample	Blood	
		Mean	CV
NA3n♀1 ×*Ca*♂1	NA3n♀1 × *Ca*♂1-1	292452.5	3.33%
	NA3n♀1 × *Ca*♂1-2	295532.6	3.41%
	NA3n♀1 × *Ca*♂1-3	288593.3	3.81%
	NA3n♀1 × *Ca*♂1-4	298962.2	3.96%
	NA3n♀1 × *Ca*♂1-5	302777.1	3.39%
	NA3n♀1 × *Ca*♂1-6	299962.3	3.68%
	NA3n♀1 × *Ca*♂1-7	304852.2	3.69%
	NA3n♀1 × *Ca*♂1-8	292914.3	3.54%
	NA3n♀1 × *Ca*♂1-9	298351.4	4.00%
	NA3n♀1 × *Ca*♂1-10	302504.3	3.63%
NA3n♀1 ×*Cc*♂1	NA3n♀1 × *Cc*♂1-1	299962.3	3.68%
	NA3n♀1 × *Cc*♂1-2	305070.6	3.51%
	NA3n♀1 × *Cc*♂1-3	293795.2	3.27%
	NA3n♀1 × *Cc*♂1-4	299307.7	3.56%
	NA3n♀1 × *Cc*♂1-5	302745.9	3.56%
	NA3n♀1 × *Cc*♂1-6	300734.9	3.49%
	NA3n♀1 × *Cc*♂1-7	307755.2	3.49%
	NA3n♀1 × *Cc*♂1-8	304311.3	3.67%
	NA3n♀1 × *Cc*♂1-9	304761.7	3.58%
	NA3n♀1 × *Cc*♂1-10	307351.4	3.55%
Control	NA3n♀1	293353.8	3.93%

Supplementary Table 8. Sex ratio of offspring from three crossed combinations.

Crossed combination	Group	♀	♂	Sex ratio (♀/♂)
NA3n♀ × *Ca*♂	NA3n♀1 × *Ca*♂1	54	0	All female
	NA3n♀2 × *Ca*♂1	48	0	
	NA3n♀3 × *Ca*♂1	67	0	
	NA3n♀4 × *Ca*♂1	49	0	
	NA3n♀5 × *Ca*♂1	55	0	
NA3n♀ × *Cc*♂	NA3n♀1 × *Cc*♂1	75	0	
	NA3n♀2 × *Cc*♂1	58	0	
	NA3n♀3 × *Cc*♂1	61	0	
	NA3n♀4 × *Cc*♂1	53	0	
	NA3n♀5 × *Cc*♂1	57	0	
Ca♀ × *Ca*♂	*Ca*♀1 × *Ca*♂1	34	36	0.94
	Ca♀2 × *Ca*♂1	29	29	1.00
	Ca♀3 × *Ca*♂1	51	48	1.06
	Ca♀4 × *Ca*♂1	45	46	0.98
	Ca♀5 × *Ca*♂1	36	34	1.06

Supplementary Table 9. Antibodies used in this study.

Name	Supplier	Catalog no	Host	Type	Dilution
anti-Sycp1	Homemade	This work	Mouse	polyclonal	1:100
anti-Sycp1	Homemade	This work	Guinea pig	polyclonal	1:100
anti-Sycp3	Homemade	This work	Mouse	polyclonal	1:100
anti-Sycp3	Homemade	This work	Rabbit	polyclonal	1:100
Anti-γH2A.X	Abcam	ab228655	Rabbit	polyclonal	1:50
Anti-Rad51	Abcam	ab88572	Mouse	polyclonal	1:100
Anti-MLH1	Abcam	ab229191	Rabbit	monoclonal	1:50
Alexa Fluor 647 Goat anti-Guinea IgG secondary Antibody	Thermo-Fisher	A-21450	Goat	polyclonal	1:300
Alexa Fluor 555 Goat anti-Mouse IgG secondary Antibody	Thermo-Fisher	A-21422	Goat	polyclonal	1:300
Alexa Fluor 488 Goat anti-Rabbit IgG secondary Antibody	Thermo-Fisher	A-11008	Goat	polyclonal	1:300
